# Prognostic value of the three-lineage cytopenia score in locally advanced nasopharyngeal carcinoma: A retrospective cohort study

**DOI:** 10.1371/journal.pone.0356005

**Published:** 2026-08-13

**Authors:** Yi Li, Zhongyi Li, Junyan He, Yang Li, Yu Duan, Dong Yang

**Affiliations:** 1 Department of Obstetrics and Gynecology, The First Affiliated Hospital, Hengyang Medical School, University of South China, Hengyang, Hunan, China; 2 Department of Oncology, The First Affiliated Hospital, Hengyang Medical School, University of South China, Hengyang, Hunan, China; All India Institute of Medical Sciences, INDIA

## Abstract

Myelosuppression is common during chemoradiotherapy for nasopharyngeal carcinoma (NPC), but the prognostic impact of multilineage cytopenia remains unclear. This retrospective study evaluated a three-lineage cytopenia score in 576 patients with locally advanced NPC. The score (0–3) was defined as the number of lineages (leukocytes, hemoglobin, platelets) below normal limits. During a median follow-up of 74 months, higher cytopenia grades were significantly associated with worse overall survival, locoregional relapse-free survival, and distant metastasis-free survival (all P < 0.001). The score was an independent prognostic factor. Compared with score 0, score 3 was associated with a 7.55-fold increased risk of death (HR = 7.545, 95%CI: 3.158–18.028) and a 21.33-fold increased risk of distant metastasis (HR = 21.333, 95%CI: 5.076–89.655). The model incorporating the three-lineage cytopenia score yielded an area under the curve (AUC) of 0.593 (95% CI: 0.533–0.652, P = 0.001), indicating modest discriminatory ability. The three-lineage cytopenia score is a simple, independent prognostic indicator for locally advanced NPC with strong predictive value for distant metastasis. It may serve as a useful adjunct to traditional staging for risk stratification.

## Introduction

Nasopharyngeal carcinoma (NPC) is a common head and neck malignancy in southern China and Southeast Asia, showing a distinct geographical clustering pattern [1]. According to Global Cancer Statistics 2022, China remains a high-incidence area for NPC, accounting for nearly half of the new cases worldwide, with a significantly higher incidence in males than in females [2]. Approximately 70% of patients are initially diagnosed at a locally advanced stage (stage III or IVa) [3].

Currently, intensity-modulated radiotherapy (IMRT) combined with chemotherapy (concurrent or induction) is the standard treatment for these patients [4]. This regimen effectively improves local tumor control and prolongs overall survival. A review by Chen et al. published in The Lancet in 2019 indicated that the application of IMRT has increased the 5-year overall survival rate for locally advanced NPC to approximately 80% [5]. However, distant metastasis remains the leading cause of treatment failure. Studies by Mao et al. have shown that about 15%–30% of patients with locally advanced NPC still develop distant metastasis after treatment [6]. Therefore, identifying high-risk patients more precisely and developing individualized treatment strategies have become a focus of clinical research.

Chemotherapy and radiotherapy, while killing tumor cells, can also damage bone marrow hematopoietic function, leading to decreased white blood cells, hemoglobin, and platelets – a condition known as myelosuppression [7]. A survey by Epstein et al. found that myelosuppression is the most common dose-limiting toxicity during chemoradiotherapy in patients with solid tumors, severely affecting treatment compliance and quality of life [8]. Previous studies have mostly focused on the prognostic impact of a single lineage of cytopenia (e.g., neutropenia) [9]. For instance, some studies have shown that chemotherapy-induced neutropenia is associated with better survival outcomes in extensive-stage small cell lung cancer, possibly reflecting indirect sensitivity to chemotherapy [10]. Conversely, other studies have observed that post-treatment thrombocytopenia is associated with poor prognosis in cancer patients [11,12]. In clinical practice, multiple cytopenias often occur simultaneously. However, whether multilineage myelosuppression (e.g., concurrent leukopenia and thrombocytopenia) has an additive negative impact on prognosis remains poorly studied.

Moreover, existing prognostic models mainly rely on tumor stage (TNM stage) and pre-treatment Epstein-Barr virus (EBV) DNA levels, rarely incorporating treatment-related toxicities such as myelosuppression [13]. The CSCO clinical guidelines published by Tang et al. in 2021 point out that although TNM stage is the primary basis for treatment decisions, patients with the same stage often have significantly different outcomes, suggesting the need for additional prognostic markers [14]. Serum biomarkers have shown potential in prognostic prediction due to their low cost and easy accessibility. Zahorec et al. systematically reviewed the application of the neutrophil-to-lymphocyte ratio in solid tumors and suggested that such markers could serve as useful adjuncts in routine clinical assessment [15]. In our preliminary research, we innovatively established a pre-treatment NLR-PLR scoring system and found it to be an independent risk factor for NPC prognosis [16].

Based on the above, we conducted this retrospective study analyzing clinical data from 576 patients with locally advanced NPC. We designed a simple scoring system—the “three-lineage cytopenia score”—which assigns 1 point for each lineage (white blood cells, hemoglobin, and platelets) below the lower limit of normal, resulting in a total score ranging from 0 to 3. We aimed to evaluate the relationship between the severity of multilineage myelosuppression and long-term survival, and to explore whether this score could serve as a new, effective prognostic tool.

## Methods

### Study population

We retrospectively analyzed 576 patients with locally advanced nasopharyngeal carcinoma (NPC) who received treatment at the First Affiliated Hospital of University of South China between January 2018 and December 2020. All patients were newly diagnosed, pathologically confirmed as NPC, and had no distant metastasis before treatment.

Inclusion criteria were: (1) pathologically confirmed NPC; (2) stage III–IVa disease according to the 8th edition of the American Joint Committee on Cancer (AJCC) staging system; (3) no prior antitumor therapy; (4) no history of other malignancies; (5) Eastern Cooperative Oncology Group (ECOG) performance status of 0–1; (6) receipt of intensity-modulated radiotherapy (IMRT) combined with induction chemotherapy and concurrent chemoradiotherapy, with completion of the planned treatment; and (7) complete clinical, laboratory, and follow-up data.

Exclusion criteria were: (1) distant metastasis detected before or during treatment; (2) severe infection or serious underlying diseases (e.g., cardiovascular or cerebrovascular diseases, uncontrolled diabetes, or mental illness); (3) history of other malignancies; and (4) failure to complete the full treatment course.

All patients underwent comprehensive evaluations before and after treatment, including head and neck magnetic resonance imaging (MRI), nasopharyngoscopy with biopsy, chest X-ray or computed tomography (CT), abdominal ultrasound or CT, and whole-body bone scan to exclude distant metastasis. This study was conducted in accordance with the Declaration of Helsinki and approved by the Ethics Committee of the First Affiliated Hospital of University of South China (Approval Number: 2025LL0415001). Because this was a retrospective study, the requirement for written informed consent was waived. The data were accessed for research purposes on 15/04/2025. All patient data were de-identified prior to analysis, the authors did not have access to any information that could identify individual participants during or after data collection.

### Clinical parameters and definitions

We collected various data from patient medical records, including imaging findings, clinical notes, and laboratory results. Pre-treatment data included age, sex, T stage, N stage, clinical stage (8th AJCC edition), pathological type, smoking history, alcohol consumption history, ECOG score, radiotherapy technique and dose, chemotherapy regimen and dose, pre-treatment albumin level, pre-treatment C-reactive protein (CRP) level, and pre- and post-treatment Epstein-Barr virus (EBV) DNA load. Laboratory parameters included white blood cell (WBC) count, hemoglobin concentration, and platelet count.

Treatment-related toxicities were graded according to the Common Terminology Criteria for Adverse Events (CTCAE) version 5.0. Based on the nadir values of blood counts during treatment, we recorded the highest grade of WBC, hemoglobin, and platelet decline for each patient.

We constructed a “three-lineage cytopenia score” as follows: for each lineage (WBC, hemoglobin, platelets) below the lower limit of normal, we assigned 1 point. The three scores were summed to obtain a total score ranging from 0 to 3. The lower limits of normal were based on our hospital laboratory standards: WBC: 4.0 × 10⁹/L; hemoglobin: 120 g/L for males and 110 g/L for females; platelets: 100 × 10⁹/L.

According to the CTCAE version 5.0, leukopenia was graded as: grade 1: < lower limit of normal (LLN) to 3.0 × 10⁹/L; grade 2: < 3.0 to 2.0 × 10⁹/L; grade 3: < 2.0 to 1.0 × 10⁹/L; grade 4: < 1.0 × 10⁹/L. For hemoglobin (anemia): grade 1: < LLN to 100 g/L; grade 2: < 100–80 g/L; grade 3: < 80–50 g/L; grade 4: < 50 g/L. For platelets (thrombocytopenia): grade 1: < LLN to 75 × 10⁹/L; grade 2: < 75–50 × 10⁹/L; grade 3: < 50–25 × 10⁹/L; grade 4: < 25 × 10⁹/L.

### Treatment regimens

All patients received standardized treatment according to the National Comprehensive Cancer Network (NCCN) guidelines. All patients underwent IMRT. Target volumes included the gross tumor volume of the nasopharynx (GTVnx), metastatic cervical lymph node volume (GTVnd), high-risk clinical target volume (CTV1), and low-risk clinical target volume (CTV2). The planning target volume (PTV) was generated by expanding the GTV or CTV by 3–5 mm. Prescribed doses were as follows: PTV-GTVnx: 68–76 Gy in 30–33 fractions; PTV-CTV1: 60–64 Gy in 30–33 fractions; PTV-CTV2: 50–54 Gy in 30–33 fractions; PTV-GTVnd: 66–70 Gy in 30–33 fractions. Dose constraints for normal tissues and organs followed the QUANTEC recommendations.

Chemotherapy regimens included concurrent chemotherapy with cisplatin 80–100 mg/m² every 3 weeks for 2–3 cycles, or cisplatin 45 mg/m² weekly for 5–6 cycles. Induction chemotherapy consisted of platinum-based doublet or triplet regimens, including gemcitabine plus cisplatin, cisplatin plus fluorouracil, paclitaxel plus cisplatin, paclitaxel plus cisplatin plus fluorouracil, and docetaxel plus cisplatin, administered every 3 weeks for 2–3 cycles. For patients unsuitable for cisplatin, other platinum agents were substituted. Maintenance therapy mainly consisted of oral capecitabine alone or combined with PD-1/PD-L1 inhibitors (e.g., camrelizumab, toripalimab, tislelizumab).

### Endpoints and follow-up

The primary endpoint was overall survival (OS), defined as the time from treatment initiation to death from any cause. Secondary endpoints were locoregional relapse-free survival (LRFS), defined as the time from treatment initiation to documented locoregional recurrence, and distant metastasis-free survival (DMFS), defined as the time from treatment initiation to documented distant metastasis.

Patients were followed up every 3 months during the first year after treatment, every 6 months during years 2–3, and annually thereafter. Follow-up was conducted through outpatient visits or telephone interviews. Treatment response was assessed according to the Response Evaluation Criteria in Solid Tumors (RECIST) version 1.1. The last follow-up date was October 31, 2025.

### Statistical analysis

All statistical analyses were performed using SPSS version 26.0 (IBM Corp., Armonk, NY, USA). A two-tailed P-value < 0.05 was considered statistically significant. Categorical variables are presented as counts (percentages) and were compared using the chi-square test or Fisher’s exact test.

Survival curves were estimated using the Kaplan-Meier method, and differences between groups were compared using the log-rank test. Variables with P ≤ 0.1 in univariate Cox regression analysis were entered into multivariate Cox proportional hazards models (Enter method) to calculate hazard ratios (HRs) and their 95% confidence intervals (CIs). Because clinical stage is highly correlated with T and N stages, only T and N stages were included in the multivariate analysis to avoid multicollinearity.

Based on the independent prognostic factors identified in the multivariate analysis, a prognostic index (linear predictor) was calculated for each patient. Patients were divided into low-, intermediate-, and high-risk groups according to tertiles of the prognostic index. Survival differences among these three groups were compared using the Kaplan-Meier method. Receiver operating characteristic (ROC) curve analysis was performed to evaluate the discriminatory ability of the model for 5-year OS, and the area under the curve (AUC) was calculated and compared with that of a model based solely on TNM stage. Subgroup analyses were conducted using stratified Cox regression, with the three-lineage cytopenia score as the sole covariate, to calculate HRs and 95% CIs in various clinical subgroups. Results were presented in a forest plot.

To address potential guarantee‑time bias, a landmark sensitivity analysis was performed. The landmark time point was set at 7 months after treatment initiation (based on the median treatment duration of approximately 4 months plus 90 days). Patients who had an event or were censored before this landmark were excluded. Kaplan‑Meier and Cox regression were then repeated using the remaining patients. The results are presented in **Supplementary Table 5** in S1 File.

A sensitivity analysis was performed by additionally adjusting for baseline white blood cell, hemoglobin, and platelet counts in the multivariate Cox model for all three endpoints. The results are presented in **Supplementary Table 6** in S1 File.

## Results

### Baseline characteristics of patients

A total of 576 patients with locally advanced nasopharyngeal carcinoma (NPC) who received curative treatment were enrolled in this study. The cohort included 437 males (75.9%) and 139 females (24.1%), with a median age of 47 years (interquartile range: 40–53). According to the 8th edition of the AJCC staging system, 314 patients (54.5%) were classified as stage III, and 262 patients (45.5%) as stage IVa. All patients received intensity-modulated radiotherapy (IMRT) combined with induction chemotherapy and concurrent chemoradiotherapy.

To evaluate the cumulative effect of multilineage myelosuppression, we developed the “three-lineage cytopenia score.” This score assigns 1 point for each lineage (white blood cell count, hemoglobin concentration, or platelet count) below the lower limit of normal, resulting in a total score ranging from 0 to 3. Based on this score, patients were divided into four groups: score 0 (n = 52, 9.0%), score 1 (n = 118, 20.5%), score 2 (n = 268, 46.5%), and score 3 (n = 138, 24.0%). The detailed distribution of each hematological parameter is presented in **Supplementary Table 1** in S1 File.

As shown in Table 1, there were no statistically significant differences among the four groups regarding sex, age, T stage, N stage, clinical stage, ECOG performance status, histological type, smoking or drinking history, pre-treatment albumin level, pre- and post-treatment EBV-DNA load, or maintenance therapy (all P > 0.05). These findings indicate good comparability among the groups. A statistically significant difference was only observed in C-reactive protein (CRP) levels (P = 0.040), with the highest proportion of elevated CRP in the score 1 group (33.9%) and the lowest in the score 0 group (17.3%).

**Table 1:** Baseline characteristics of patients stratified by the three-lineage cytopenia score.

Characteristic	Total Cohort (n = 576, %)	Three-Lineage Cytopenia Score				P-value
		0 (n = 52, %)	1 (n = 118, %)	2 (n = 268, %)	3 (n = 138, %)	
**Gender**						0.252
Male	437(75.9)	45(86.5)	86(72.9)	200(74.6)	106(76.8)	
Female	139(24.1)	7(13.5)	32(27.1)	68(25.4)	32(23.2)	
**Age (years)**						0.087
<47	283(49.1)	29(55.8)	61(51.7)	138(51.5)	55(39.9)	
≥47	293(50.9)	23(44.2)	57(48.3)	130(48.5)	83(60.1)	
**8**^**th**^ **T stage**						0.242
T1-T2	104(18.1)	8(15.4)	18(15.3)	51(19.0)	27(19.6)	
T3	278(48.3)	33(63.4)	54 (45.7)	130(48.5)	61(44.2)	
T4	194(33.6)	11(21.2)	46(39.0)	87(32.5)	50(36.2)	
**8**^**th**^ **N stage**						0.803
N0-N1	132(22.9)	14(26.9)	30(25.4)	63(23.5)	25(18.1)	
N2	353(61.3)	29(55.8)	70(59.3)	163(60.8)	91(65.9)	
N3	91(15.8)	9(17.3)	18(15.3)	42(15.7)	22(15.9)	
**Clinical stage**						0.432
III	314(54.5)	33(63.5)	60(50.8)	149(55.6)	72(52.2)	
IVa	262(45.5)	19(36.5)	58(49.2)	119(44.4)	66(47.8)	
**ECOG PS**						0.685
0	276(47.9)	25(48.1)	59(50.0)	132(49.3)	60(43.5)	
1	300(52.1)	27(51.9)	59(50.0)	136(50.7)	78(56.5)	
**Histology type**						0.979
NKUC	560(97.2)	51 (98.1)	114(96.6)	261(97.4)	134(97.1)	
others	16(2.8)	1(1.9)	4(3.4)	7(2.6)	4(2.9)	
**Drinking history**						0.793
No	492(85.4)	42(80.8)	101(85.6)	231(86.2)	118(85.5)	
Yes	84(14.6)	10(19.2)	17(14.4)	37(13.8)	20(14.5)	
**Smoking history**						0.774
No	377(65.5)	31(59.6)	76(64.4)	177(66.0)	93(67.4)	
Yes	199(34.5)	21(40.4)	42(35.6)	91(34.0)	45(32.6)	
**Albumin (g/L)**						0.067
<43.4 g/L	275(47.7)	23(44.2)	68(57.6)	116(43.3)	68(49.3)	
≥43.4 g/L	301(52.3)	29(55.8)	50(42.4)	152(56.7)	70(50.7)	
**CRP**						0.040
Normal	433(75.2)	43(82.7)	78(66.1)	202(75.4)	110(79.7)	
Elevated	143(24.8)	9(17.3)	40(33.9)	66(24.6)	28(20.3)	
**Pre-treatment EBV-DNA**						0.424
<400copies/mL	275(47.7)	24(46.2)	64(54.2)	126(47.0)	61(44.2)	
≥400copies/mL	301(52.3)	28(53.8)	54(45.8)	142 (53.0)	77(55.8)	
**Post-treatment EBV-DNA**						0.531
<400copies/mL	411(71.4)	39(75.0)	87(73.7)	193(72.0)	92(66.7)	
≥400copies/mL	165(28.6)	13(25.0)	31(26.3)	75(28.0)	46(33.3)	
**Maintenance therapy**						0.307
No	287(49.8)	29(55.8)	66(55.9)	128(47.8)	64(46.4)	
Yes	289(50.2)	23(44.2)	52(44.1)	140(52.2)	74(53.6)	

Note: The three-lineage cytopenia score is defined as the sum of the number of lineages (white blood cells, hemoglobin, platelets) below the lower limit of normal, with each lineage counting as 1 point. Data are presented as n (%). P-values were calculated using the chi-square test or Fisher’s exact test for comparisons among the four groups. CRP, C-reactive protein; NKUC, non-keratinizing undifferentiated carcinoma.

### Survival outcomes

**Fig 1:**
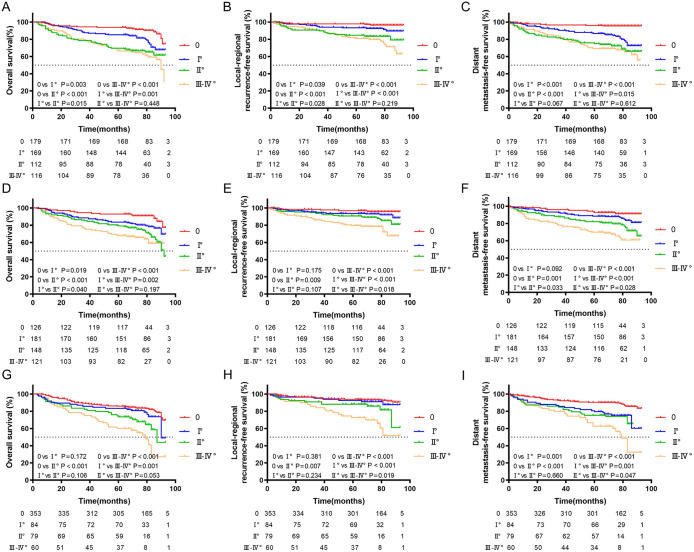
Kaplan-Meier curves for overall survival (OS), locoregional relapse-free survival (LRFS), and distant metastasis-free survival (DMFS) according to post-treatment cytopenia grades. (A–C) Leukopenia grade; (D–F) Hemoglobin decline grade; (G–I) Thrombocytopenia grade.

**Fig 2:**
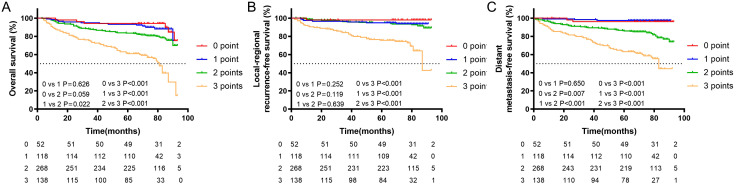
Kaplan-Meier curves for OS (A), LRFS (B), and DMFS (C) according to the three-lineage cytopenia score. The score is defined as the number of lineages (leukocytes, hemoglobin, platelets) below the lower limit of normal (range 0–3). Note: Detailed survival rates and pairwise comparison results are available in **Supplementary Table 2** in S1 File.

The median follow-up time was 74 months (range: 66–83 months). During follow-up, 145 patients died, 65 experienced local recurrence, and 111 developed distant metastasis. Kaplan-Meier survival analysis showed that the grades of leukopenia, hemoglobin decline, and thrombocytopenia were all significantly associated with 5-year overall survival (OS), locoregional relapse-free survival (LRFS), and distant metastasis-free survival (DMFS) (all log-rank P < 0.001). The corresponding survival curves are presented in Fig 1 (Fig 1A–C**: leukopenia grade with OS, LRFS, and DMFS;** Fig 1D–F**: hemoglobin decline grade;** Fig 1G–I**: thrombocytopenia grade)**. To further evaluate the cumulative effect of multilineage myelosuppression, we developed the “three-lineage cytopenia score” (assigning 1 point for each lineage below the lower limit of normal). This score was also significantly associated with all three survival endpoints (P < 0.001), and its survival curves are shown in Fig 2 (Fig 2A–C).

Leukopenia and prognosis: As the grade of leukopenia increased, patient survival rates decreased progressively. For OS, the 5-year OS rates were 88.8%, 77.5%, 65.2%, and 58.6% for grades 0, I°, II, and III, respectively (P < 0.001). For LRFS, the 5-year LRFS rates were 96.6%, 91.7%, 83.9%, and 76.7% (P < 0.001). For DMFS, the 5-year DMFS rates were 95.5%, 80.5%, 71.4%, and 67.2% (P < 0.001). Pairwise comparisons revealed no significant differences between grade II and grade III for any endpoint (OS: P = 0.448; LRFS: P = 0.219; DMFS: P = 0.612), while all other between-group differences were statistically significant.

Hemoglobin decline and prognosis: Hemoglobin decline also showed significant prognostic stratification. For OS, the 5-year OS rates were 88.9%, 78.5%, 67.6%, and 63.6% for grades 0, I, II, and III, respectively (P < 0.001). For LRFS, the 5-year LRFS rates were 96.0%, 92.3%, 87.2%, and 77.7% (P < 0.001). For DMFS, the 5-year DMFS rates were 92.1%, 85.6%, 76.4%, and 66.9% (P < 0.001). Pairwise comparisons showed a significant difference between grade I and grade 0 for OS (P = 0.019), and between grade II and grade III for both LRFS (P = 0.018) and DMFS (P = 0.028).

Thrombocytopenia and prognosis: Thrombocytopenia had the most pronounced impact on prognosis. For OS, the 5-year OS rates were 80.7%, 76.2%, 65.8%, and 50.0% for grades 0, I, II, and III, respectively (P < 0.001). For LRFS, the 5-year LRFS rates were 92.6%, 90.5%, 84.8%, and 68.3% (P < 0.001). For DMFS, the 5-year DMFS rates were 87.8%, 75.0%, 73.4%, and 56.7% (P < 0.001). Pairwise comparisons indicated that grade III differed significantly or marginally significantly from all other groups across the three endpoints (OS: III vs. II, P = 0.053; LRFS: III vs. II, P = 0.019; DMFS: III vs. II, P = 0.047), suggesting that severe thrombocytopenia is the strongest marker of poor prognosis.

Three-lineage cytopenia score and prognosis: The three-lineage cytopenia score was strongly associated with survival outcomes. The association was most pronounced for patients with a score of 3 (involvement of all three lineages), who had substantially worse OS, LRFS, and DMFS compared to those with lower scores. However, there was no consistent monotonic dose–response relationship, as differences between scores 0, 1, and 2 were either non‑significant or inconsistent across endpoints (see pairwise comparisons below). For OS, the 5-year OS rates were 88.5%, 89.0%, 78.4%, and 50.7% for scores 0, 1, 2, and 3, respectively (P < 0.001). For LRFS, the 5-year LRFS rates were 98.1%, 94.1%, 92.9%, and 72.5% (P < 0.001). For DMFS, the 5-year DMFS rates were 96.2%, 97.5%, 81.3%, and 59.4% (P < 0.001). Pairwise comparisons showed that the score 3 group differed significantly from all other groups across all endpoints (all P < 0.001). For OS, the difference between score 0 and score 1 was not significant (P = 0.626), and the difference between score 0 and score 2 was borderline significant (P = 0.059). For LRFS, score 0 did not differ significantly from score 1 (P = 0.252) or score 2 (P = 0.119). For DMFS, score 0 and score 1 were not significantly different (P = 0.650), but score 0 differed significantly from score 2 (P = 0.007), and score 1 differed significantly from score 2 (P < 0.001). These results indicate that multilineage myelosuppression, especially involving all three lineages, is a strong predictor of poor prognosis.

In the landmark sensitivity analysis with a 7‑month landmark, 36 patients were excluded from the OS analysis (6.3%), leaving 540 patients with 82 events. The three‑lineage cytopenia score, analyzed as a continuous variable, remained significantly associated with OS (HR = 2.182 per 1‑point increase; 95% CI: 1.741–2.734; P < 0.001). Consistent results were observed for LRFS (HR = 2.994; 95% CI: 2.053–4.368; P < 0.001) and DMFS (HR = 3.052; 95% CI: 2.270–4.103; P < 0.001; Supplementary Table 5 in S1 File).

### Multivariate cox regression analysis

To determine whether the post-treatment three-lineage cytopenia score was an independent prognostic factor for patients with locally advanced nasopharyngeal carcinoma, we first performed univariate Cox regression analysis (**Supplementary Table 3** in S1 File). Variables with P ≤ 0.1 in the univariate analysis were subsequently included in the multivariate Cox proportional hazards model. Multivariate analyses were conducted for 5-year overall survival (OS), locoregional relapse-free survival (LRFS), and distant metastasis-free survival (DMFS), with results presented in Table 2.

**Table 2:** Multivariate Cox regression analysis of prognostic factors in NPC patients.

Characteristics	5-year OS		5-year LRRFS		5-year DMFS	
	HR (95%CI)	*P*-Value	HR (95%CI)	*P*-Value	HR (95%CI)	*P*-Value
**Age (years)**						
<47	Reference		Reference		-	
≥47	1.812(0.1.288-2.548)	0.001	2.058(1.222-3.466)	0.007	-	-
**8^th^ T stage**		0.004		<0.001		-
T1-2	Reference		Reference		-	
T3	1.597(0.937-2.719)	0.085	2.842(0.828-9.748)	0.0097	-	-
T4	2.313(1.369-3.910)	0.002	6.931(2.083-23.063)	0.002	-	-
**8^th^ N stage**		<0.001				<0.001
N0-N1	Reference		-		Reference	
N2	1.246(0.767-2.024)	0.374	-	-	1.576(0.818-3.035)	0.174
N3	3.518(2.052-6.029)	<0.001	-	-	6.496(3.172-13.304)	<0.001
**ECOG**						
0	Reference		Reference		Reference	
1	2.115(1.453-3.079)	<0.001*	2.546(1.442-4.495)	0.001	1.994(1.305-3.045)	0.001
**Histology type**						
NKUC	Reference		Reference		-	
Others	1.755(0.804-3.832)	0.158	4.001(1.688-9.483)	0.002	-	-
**Drinking history**						
No	-		-		Reference	
Yes	-	-	-	-	2.102(1.298-3.404)	0.003
**Albumin**						
<43.4g/L	Reference		Reference		-	
≥43.4g/L	0.675(0.482-0.947)	0.023	0.755(0.456-1.248)	0.273	-	-
**Pre-treatment EBV-DNA**						
<400copies/mL	Reference		Reference		Reference	
≥400copies/mL	1.064(0.664-1.705)	0.797	1.168(0.557-2.451)	0.681	1.119(0.659-1.899)	0.677
**Post-treatment EBV-DNA**						
<400copies/mL	Reference		Reference		Reference	
≥400copies/mL	2.046(1.284-3.261)	0.003	2.650(1.321-5.314)	0.006	1.969 (1.162-3.338)	0.012
**Maintenance therapy**						
No	-		-		Reference	
Yes	-	-	-	-	0.622(0.424-0.912)	0.015
**Three-lineage cytopenia score**		<0.001		<0.001		<0.001
0	Reference		Reference		Reference	
1	1.325(0.489-3.589)	0.580	3.130(0.370-26.466)	0.295	1.362(0.246-7.535)	0.724
2	2.893(1.218-6.873)	0.016	4.516(0.589-34.658)	0.147	7.871(1.897-32.662)	0.004
3	7.545(3.158-18.028)	<0.001	19.596(2.567-149.582)	0.004	21.333(5.076-89.655)	<0.001

Note: Only variables with P ≤ 0.1 in univariate analysis were included in the multivariate model. Clinical stage was not included due to high correlation with T stage and N stage. HR, hazard ratio; CI, confidence interval; NKUC, non-keratinizing undifferentiated carcinoma; EBV, Epstein-Barr virus.

After adjusting for potential confounders including age, T stage, N stage, ECOG performance status, histological type, drinking history, pre- and post-treatment EBV-DNA load, and maintenance therapy, the three-lineage cytopenia score remained an independent prognostic factor for all three survival endpoints (all overall P < 0.001).

**Independent prognostic factors for OS:** Compared with a score of 0, a score of 2 was associated with a 2.89-fold increased risk of death (HR = 2.893, 95% CI: 1.218–6.873, P = 0.016), and a score of 3 was associated with a 7.55-fold increased risk (HR = 7.545, 95% CI: 3.158–18.028, P < 0.001). Other independent prognostic factors included age ≥ 47 years (HR = 1.812, 95% CI: 1.288–2.548, P = 0.001), T4 stage (HR = 2.313, 95% CI: 1.369–3.910, P = 0.002), N3 stage (HR = 3.518, 95% CI: 2.052–6.029, P < 0.001), ECOG 1 (HR = 2.115, 95% CI: 1.453–3.079, P < 0.001), and post-treatment EBV-DNA ≥ 400 copies/mL (HR = 2.046, 95% CI: 1.284–3.261, P = 0.003). Albumin ≥43.4 g/L was a protective factor (HR = 0.675, 95% CI: 0.482–0.947, P = 0.023).

**Independent prognostic factors for LRFS:** Patients with a score of 3 had a 19.60-fold increased risk of local recurrence compared to those with a score of 0 (HR = 19.596, 95% CI: 2.567–149.582, P = 0.004). Other significant factors included age ≥ 47 years (HR = 2.058, 95% CI: 1.222–3.466, P = 0.007), T3 stage (HR = 2.842, 95% CI: 0.828–9.748, P = 0.097, borderline significant), T4 stage (HR = 6.931, 95% CI: 2.083–23.063, P = 0.002), ECOG 1 (HR = 2.546, 95% CI: 1.442–4.495, P = 0.001), other histological types (HR = 4.001, 95% CI: 1.688–9.483, P = 0.002), and post-treatment EBV-DNA ≥ 400 copies/mL (HR = 2.650, 95% CI: 1.321–5.314, P = 0.006).

**Independent prognostic factors for DMFS:** The three-lineage cytopenia score showed particularly strong predictive value for distant metastasis. Compared with a score of 0, a score of 2 was associated with a 7.87-fold increased risk of distant metastasis (HR = 7.871, 95% CI: 1.897–32.662, P = 0.004), and a score of 3 was associated with a 21.33-fold increased risk (HR = 21.333, 95% CI: 5.076–89.655, P < 0.001). Other independent prognostic factors included N3 stage (HR = 6.496, 95% CI: 3.172–13.304, P < 0.001), ECOG 1 (HR = 1.994, 95% CI: 1.305–3.045, P = 0.001), drinking history (HR = 2.102, 95% CI: 1.298–3.404, P = 0.003), and post-treatment EBV-DNA ≥ 400 copies/mL (HR = 1.969, 95% CI: 1.162–3.338, P = 0.012). Maintenance therapy was identified as a protective factor (HR = 0.622, 95% CI: 0.424–0.912, P = 0.015).

In summary, multivariate analysis confirmed that the post-treatment three-lineage cytopenia score is a strong independent predictor of poor prognosis in patients with locally advanced nasopharyngeal carcinoma, with particularly pronounced predictive value for distant metastasis risk.

### Prognostic model based on the three-lineage cytopenia score and subgroup analysis

#### Development and evaluation of the prognostic model.

**Fig 3:**
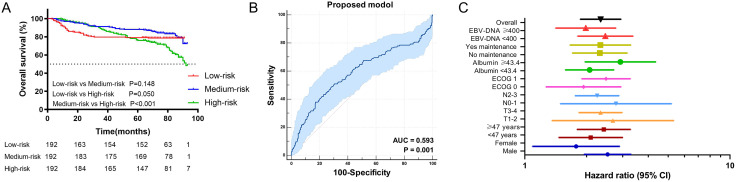
Evaluation of the prognostic model based on the three-lineage cytopenia score. (A) Kaplan-Meier survival curves for overall survival according to risk stratification by the prognostic index tertiles (low-risk, intermediate-risk, and high-risk groups). (B) Receiver operating characteristic (ROC) curve for the model incorporating the three-lineage cytopenia score (AUC = 0.593, 95% CI: 0.533–0.652). (C) Forest plot of subgroup analysis for the prognostic value of the three-lineage cytopenia score on overall survival. Squares represent the hazard ratio (HR) per 1-point increase in the score within each subgroup, with horizontal lines indicating 95% confidence intervals. The vertical dashed line denotes HR = 1, and the diamond represents the overall HR for the entire cohort.

Based on the independent prognostic factors for OS identified by multivariate Cox regression analysis, a prognostic index (linear predictor) was calculated for each patient. Patients were then divided into three groups according to the tertiles of this index: low-risk (n = 192), intermediate-risk (n = 192), and high-risk (n = 192). Kaplan-Meier survival analysis showed that the 5-year OS rates for the low-, intermediate-, and high-risk groups were 78.6%, 82.8%, and 62.5%, respectively (Fig 3A). Of note, the intermediate-risk group had a slightly higher 5‑year OS rate (82.8%) than the low‑risk group (78.6%), although the difference was not statistically significant (P = 0.148). This suggests that the prognostic index could not reliably distinguish between low‑ and intermediate‑risk patients, but it successfully identified the high‑risk group as having the worst prognosis. The high-risk group had significantly worse survival than both the intermediate-risk group (P < 0.001) and the low-risk group (P = 0.050), indicating that the prognostic index effectively identifies patients with the poorest outcomes.

The discriminatory ability of the model for predicting 5-year OS was further assessed using receiver operating characteristic (ROC) curve analysis (Fig 3B). The model incorporating the three-lineage cytopenia score yielded an area under the curve (AUC) of 0.593 (95% CI: 0.533–0.652, P = 0.001), indicating modest discriminatory ability. Of note, the TNM‑only model had an AUC below 0.5 (data not shown), suggesting poor performance of TNM staging alone in this cohort, which may be due to sample characteristics. Therefore, we did not perform a formal comparison. These findings suggest that adding the three-lineage cytopenia score may provide additional prognostic information for patients with locally advanced nasopharyngeal carcinoma, although the modest AUC indicates that further refinement is needed.

### Subgroup analysis

To assess whether the prognostic value of the three-lineage cytopenia score for OS was consistent across different clinical subgroups, we performed stratified Cox regression analyses. As shown in Fig 3C and **Supplementary Table 4** in S1 File, the three-lineage cytopenia score (as a continuous variable) maintained its significant prognostic effect in nearly all subgroups. The hazard ratio (HR) per 1-point increase in the score was 2.533 (95% CI: 1.940–3.306) in males and 1.777 (95% CI: 1.088–2.904) in females. In patients aged <47 and ≥47 years, the HRs were 2.095 (95% CI: 1.460–3.006) and 2.426 (95% CI: 1.793–3.282), respectively. For T1–2 and T3–4 stages, HRs were 2.688 (95% CI: 1.354–5.334) and 2.340 (95% CI: 1.830–2.993). For N0–1 and N2–3 stages, HRs were 2.782 (95% CI: 1.487–5.205) and 2.251 (95% CI: 1.755–2.887). In patients with ECOG 0 and 1, HRs were 1.933 (95% CI: 1.265–2.955) and 2.488 (95% CI: 1.889–3.279). For albumin <43.4 g/L and ≥43.4 g/L, HRs were 2.070 (95% CI: 1.571–2.728) and 2.922 (95% CI: 1.959–4.359). In patients without and with maintenance therapy, HRs were 2.321 (95% CI: 1.701–3.168) and 2.335 (95% CI: 1.655–3.295). For post-treatment EBV-DNA < 400 copies/mL and ≥400 copies/mL, HRs were 2.469 (95% CI: 1.805–3.378) and 1.982 (95% CI: 1.409–2.788).

The 95% confidence intervals of the HRs for all subgroups overlapped considerably and all included the overall HR of 2.341 (95% CI: 1.858–2.951), indicating no significant interaction. These results demonstrate that the three-lineage cytopenia score is a robust and consistent prognostic predictor across patients with diverse clinical characteristics. Detailed subgroup analysis data are provided in **Supplementary Table 4** in S1 File.

## Discussion

In this study, we observed a clear trend: more severe post-treatment myelosuppression was associated with poorer long-term survival in patients with locally advanced nasopharyngeal carcinoma. Our main findings can be summarized as follows. First, the degree of decline in leukocytes, hemoglobin, and platelets was each significantly associated with 5-year overall survival (OS), locoregional relapse-free survival (LRFS), and distant metastasis-free survival (DMFS). Survival rates decreased progressively with increasing grades of cytopenia. Second, the proposed “three-lineage cytopenia score” remained a strong independent predictor for all three survival endpoints after adjusting for established prognostic factors such as age, tumor stage, and performance status. Notably, patients with involvement of all three lineages (score 3) had a more than 7-fold increased risk of death and a 21-fold increased risk of distant metastasis compared to those without myelosuppression. Third, incorporating this score into the traditional TNM staging system showed modest improvement in the model’s discriminatory ability compared to TNM staging alone. Fourth, we validated the prognostic consistency of this score across various patient subgroups—whether stratified by sex, age, tumor stage, or other clinical characteristics, a higher score consistently indicated worse outcomes. These findings offer new insights into the clinical significance of myelosuppression.

Previous studies on the relationship between myelosuppression and prognosis in nasopharyngeal carcinoma have largely focused on single hematopoietic lineages. For instance, a 2016 study by Huang et al. found that patients who developed neutropenia during induction chemotherapy had significantly lower 5-year OS and disease-free survival rates than those who did not [17]. Interestingly, they also observed that even grade 1–2 neutropenia was associated with poorer outcomes, and the degree of deterioration did not differ significantly between patients with grade 1–2 and those with grade 3–4 neutropenia. This finding closely parallels our observations regarding leukopenia: we found no statistically significant difference in survival between patients with grade II and grade III leukopenia. This suggests that once a certain threshold of leukopenia is reached (grade II or higher), its adverse impact on prognosis may plateau, with further escalation in grade conferring no additional detriment.

However, these studies did not address an important question: does simultaneous decline in multiple hematopoietic lineages lead to worse outcomes? This is where our study offers innovation. By integrating information from all three lineages into a composite score, we clearly demonstrated that as the number of affected lineages increased, the risks of death and distant metastasis escalated progressively. Patients with all three lineages involved had substantially higher risks of death (7.5-fold) and distant metastasis (21.3-fold) than those with only one or two affected lineages—a phenomenon not previously reported. This finding suggests that multilineage myelosuppression represents more than the simple sum of individual toxicities; it may reflect more profound systemic injury or indicate that the patient’s bone marrow reserve is severely compromised.

What might explain the strong association between multilineage myelosuppression and poor prognosis? Several mechanisms may be involved. First, myelosuppression directly impacts treatment delivery. When severe myelosuppression occurs, clinicians often must reduce chemotherapy doses or delay subsequent cycles, potentially compromising treatment intensity and long-term efficacy [18]. A predictive model study by Wang et al. published in 2025 confirmed that patients experiencing myelosuppression during chemoradiotherapy had significantly lower treatment completion rates and worse survival outcomes than those without myelosuppression [13]. In our cohort, although the proportion of patients receiving maintenance therapy was similar across groups, those with all three lineages affected had a 5-year OS rate of only 50.7%, far lower than the 88.5% observed in patients without myelosuppression. This suggests that even with standard treatment, these patients are physiologically less resilient, leading to poorer outcomes.

Second, the bone marrow serves as the factory for immune cells, including neutrophils and lymphocytes. Suppression of bone marrow function inevitably compromises immune capacity. Numerous recent studies have linked an elevated post-treatment neutrophil-to-lymphocyte ratio (NLR) to poor prognosis in nasopharyngeal carcinoma [16,19]. A meta-analysis by Zhao et al. demonstrated that a high NLR is an independent adverse prognostic factor for both OS and progression-free survival in nasopharyngeal carcinoma (HR = 1.76, 95% CI: 1.47–2.12) [20]. Similarly, Takenaka et al. confirmed that a high NLR is significantly associated with worse OS, disease-specific survival, and DMFS [21]. Although our three-lineage cytopenia score does not directly incorporate lymphocyte counts, patients with multilineage involvement likely also experience lymphopenia, thereby compromising their ability to mount an effective anti-tumor immune response—particularly against distant metastases. This aligns with our observation that the score’s impact on distant metastasis risk (HR = 21.33) was substantially greater than its impact on local recurrence risk (HR = 19.60).

Finally, myelosuppression may serve as a “signal” reflecting poor baseline health status [22]. Patients with poor nutritional status or chronic inflammation may be more susceptible to myelosuppression. A 2025 study by Cetintepe et al. found that low albumin levels were significantly associated with bone marrow metastasis in patients with solid tumors, highlighting the importance of nutritional status in maintaining bone marrow function [23]. Our data also showed differences in pre-treatment C-reactive protein levels among patients with different scores, indirectly supporting this notion.

Regarding innovation, our study has several notable strengths. First, we proposed a novel yet simple indicator—the “three-lineage cytopenia score”—that can be easily calculated from routine complete blood counts without the need for additional testing, making it readily applicable in clinical practice. This aligns with the recent trend of integrating serum biomarkers into prognostic models [16,24]. Second, we demonstrated that this score has particularly strong predictive value for distant metastasis, the leading cause of treatment failure in nasopharyngeal carcinoma. Early identification of high-risk patients (e.g., those with all three lineages involved) could enable clinicians to intensify systemic therapy, such as extending maintenance chemotherapy or initiating immunotherapy earlier [25]. Third, by comparing our score-based model with the traditional TNM staging system, we showed that incorporating treatment-related toxicity adds value to existing prognostic frameworks. Although the discriminatory ability of our model (AUC = 0.593) is not yet optimal, it significantly outperformed TNM staging alone (AUC = 0.372). This at least suggests that integrating treatment-related toxicity into prognostic assessment is a worthwhile avenue for exploration. Yang et al. recently used dual-source CT with calcium-suppression imaging to assess bone marrow function and successfully predict chemotherapy-induced myelosuppression risk, offering a novel imaging-based approach for evaluating bone marrow reserve [26]. Fourth, we validated the prognostic consistency of our score across diverse patient populations, enhancing its credibility and potentially increasing clinicians’ confidence in using it.

Nevertheless, this study has several limitations. First, this was a retrospective study conducted at a single institution, which may have introduced selection bias [27]. Although we adjusted for various known confounders, unmeasured factors may still have influenced the results. To address potential guarantee‑time bias, we performed a landmark sensitivity analysis (Supplementary Table 5 in S1 File) and also adjusted for baseline blood counts (Supplementary Table 6 in S1 File); these analyses supported the robustness of our main findings. Detailed information on G‑CSF use, transfusion support, and chemotherapy dose modifications was not uniformly available in this retrospective dataset, and these unmeasured factors may have influenced the observed associations. Future multicenter studies with larger cohorts are needed to validate our findings. Second, we assessed myelosuppression based on nadir values during treatment, without considering the timing, duration, or recovery pattern of cytopenia. The dynamic trajectory of myelosuppression may contain more prognostic information than a single nadir value [28]. Future studies could employ dynamic analytical methods, such as trajectory modeling, to identify particularly hazardous patterns of cytopenia. Third, the discriminatory ability of our predictive model (AUC = 0.593) leaves substantial room for improvement. Combining this score with novel biomarkers, such as dynamic changes in EBV-DNA or radiomic features, could potentially yield more accurate models [29]. Finally, our data were collected before the widespread adoption of immunotherapy in nasopharyngeal carcinoma and do not reflect this aspect. Given that immunotherapy is now a mainstay of treatment, the relationship between immune-related toxicities and prognosis warrants further investigation [30]. A study by Weidhaas et al. identified specific genetic markers that predict both toxicity and response to immunotherapy, providing new avenues for personalized treatment [31].

What are the implications of this study for future clinical practice and research? From a clinical perspective, the three-lineage cytopenia score offers a simple and inexpensive tool for identifying patients at potentially higher risk after chemoradiotherapy. When encountering patients with all three lineages affected, clinicians may consider closer follow-up, although prospective validation is needed before clinical implementation. From a research perspective, our findings suggest that treatment-related toxicities should not be viewed merely as “side effects” but may themselves serve as important biological signals [32]. Future research could explore the molecular mechanisms underlying multilineage myelosuppression, such as investigating genetic characteristics or immune system abnormalities in these patients. Elucidating these mechanisms could lead to targeted interventions aimed at mitigating the adverse effects of myelosuppression and ultimately improving outcomes for this high-risk population.

In conclusion, this study is the first to propose and validate the three-lineage cytopenia score as an independent prognostic indicator for patients with locally advanced nasopharyngeal carcinoma. This simple, practical score effectively identifies those at highest risk, particularly patients with involvement of all three lineages, and demonstrates promising predictive value for distant metastasis. Adding this score to the traditional TNM staging system may provide additional prognostic information. Although many questions remain to be addressed in future studies, we believe this work provides a potential tool for risk stratification in nasopharyngeal carcinoma.

## Supporting information

S1 File Supplementary Table. Supporting Information Tables S1–S6: **Supplementary Table 1.** Distribution of post-treatment myelosuppression indicators in the entire cohort. **Supplementary Table 2.** Univariate analysis of post-treatment myelosuppression grades and 5-year survival rates. **Supplementary Table 3.** Univariate Cox regression analysis of prognostic factors in NPC patients. **Supplementary Table 4.** Subgroup analysis of the three-lineage cytopenia score for overall survival. **Supplementary Table 5.** Landmark sensitivity analysis (7 months after treatment initiation) for overall survival (OS), locoregional relapse‑free survival (LRFS), and distant metastasis‑free survival (DMFS) by cytopenia grade and three‑lineage cytopenia score. **Supplementary Table 6.** Sensitivity analysis for OS, LRFS, and DMFS adjusting for baseline blood counts (three‑lineage cytopenia score as a continuous variable).
(DOCX)

## References

[1] GBD 2021 Causes of Death Collaborators. Global burden of 288 causes of death and life expectancy decomposition in 204 countries and territories and 811 subnational locations, 1990-2021: a systematic analysis for the Global Burden of Disease Study 2021. Lancet. 2024;403(10440):2100–32. doi: 10.1016/S0140-6736(24)00367-2 38582094 PMC11126520

[2] Bray F, Laversanne M, Sung H, et al. Global cancer statistics 2022: GLOBOCAN estimates of incidence and mortality worldwide for 36 cancers in 185 countries. CA Cancer J Clin. 2024;74(3):229–63. doi: 10.3322/caac.21834 38572751

[3] Mao Y-P, Xie F-Y, Liu L-Z, Sun Y, Li L, Tang L-L, et al. Re-evaluation of 6th edition of AJCC staging system for nasopharyngeal carcinoma and proposed improvement based on magnetic resonance imaging. Int J Radiat Oncol Biol Phys. 2009;73(5):1326–34. doi: 10.1016/j.ijrobp.2008.07.062 19153016

[4] Colevas AD, Cmelak AJ, Pfister DG, et al. NCCN Guidelines® Insights: Head and Neck Cancers, Version 2.2025. J Natl Compr Canc Netw. 2025;23(2):2–11. doi: 10.6004/jnccn.2025.0007 39938471

[5] Chen Y-P, Chan ATC, Le Q-T, Blanchard P, Sun Y, Ma J. Nasopharyngeal carcinoma. Lancet. 2019;394(10192):64–80. doi: 10.1016/S0140-6736(19)30956-0 31178151

[6] Mao Y-P, Tang L-L, Chen L, Sun Y, Qi Z-Y, Zhou G-Q, et al. Prognostic factors and failure patterns in non-metastatic nasopharyngeal carcinoma after intensity-modulated radiotherapy. Chin J Cancer. 2016;35(1):103. doi: 10.1186/s40880-016-0167-2 28031050 PMC5192583

[7] Barreto JN, McCullough KB, Ice LL, Smith JA. Antineoplastic agents and the associated myelosuppressive effects: a review. J Pharm Pract. 2014;27(5):440–6. doi: 10.1177/0897190014546108 25147158

[8] Epstein RS, Aapro MS, Basu Roy UK, Salimi T, Krenitsky J, Leone-Perkins ML, et al. Patient Burden and Real-World Management of Chemotherapy-Induced Myelosuppression: Results from an Online Survey of Patients with Solid Tumors. Adv Ther. 2020;37(8):3606–18. doi: 10.1007/s12325-020-01419-6 32642965 PMC7340862

[9] Ma XH, Liang CX, Jiang DX, et al. Clinical observation of TPF induction chemotherapy or PF induction chemotherapy combined with concurrent chemoradiotherapy in the treatment of locally advanced nasopharyngeal carcinoma. Chinese Journal of Cancer. 2016;26(12):1018–24. doi: 10.19401/j.cnki.1007-3639.2016.12.009

[10] Miyagi T, Tsuji D, Kawasakai Y, Ishikawa H, Tanaka R, Nakao M, et al. Chemotherapy-induced neutropenia as a prognostic factor in patients with extensive-stage small cell lung cancer. Eur J Clin Pharmacol. 2023;79(3):407–14. doi: 10.1007/s00228-023-03451-1 36645467

[11] Pang Q, Qu K, Zhang J-Y, Song S-D, Liu S-S, Tai M-H, et al. The Prognostic Value of Platelet Count in Patients With Hepatocellular Carcinoma: A Systematic Review and Meta-Analysis. Medicine (Baltimore). 2015;94(37):e1431. doi: 10.1097/MD.0000000000001431 26376382 PMC4635796

[12] Wang Q, Ye T, Chen H-L, Zhang X-G, Zhang L-Z. Correlation between intensity modulated radiotherapy and bone marrow suppression in breast cancer. Eur Rev Med Pharmacol Sci. 2016;20(1):75–81. 26813456

[13] Wang R-H, Jiang D-Y, Lu J, Xun L-X, Wang F, Shao Q-Q, et al. Development of a Predictive Model for the Risk of Myelosuppression in Patients With Nasopharyngeal Carcinoma Undergoing Chemoradiotherapy. Clin Med Insights Oncol. 2025;19:11795549251381678. doi: 10.1177/11795549251381678 41049386 PMC12489199

[14] Tang L-L, Chen Y-P, Chen C-B, Chen M-Y, Chen N-Y, Chen X-Z, et al. The Chinese Society of Clinical Oncology (CSCO) clinical guidelines for the diagnosis and treatment of nasopharyngeal carcinoma. Cancer Commun (Lond). 2021;41(11):1195–227. doi: 10.1002/cac2.12218 34699681 PMC8626602

[15] Zahorec R. Neutrophil-to-lymphocyte ratio, past, present and future perspectives. Bratisl Lek Listy. 2021;122(7):474–88. doi: 10.4149/BLL_2021_078 34161115

[16] Yang D, Li P, Meng Z, Hu X, Huang Z, Huang H, et al. Combined pretreatment neutrophil-lymphocyte ratio and platelet-lymphocyte ratio predicts survival and prognosis in patients with non-metastatic nasopharyngeal carcinoma: a retrospective study. Sci Rep. 2024;14(1):9898. doi: 10.1038/s41598-024-59131-2 38688967 PMC11061272

[17] Xing X, Zhou X, Yang Y, Li Y, Hu C, Shen C. The impact of body composition parameters on severe toxicities in patients with locoregionally advanced nasopharyngeal carcinoma undergoing neoadjuvant chemotherapy. Ann Transl Med. 2021;9(14):1180. doi: 10.21037/atm-21-3412 34430621 PMC8350723

[18] Oliva M, Huang SH, Taylor R, Su J, Xu W, Hansen AR, et al. Impact of cumulative cisplatin dose and adjuvant chemotherapy in locally-advanced nasopharyngeal carcinoma treated with definitive chemoradiotherapy. Oral Oncol. 2020;105:104666. doi: 10.1016/j.oraloncology.2020.104666 32272384

[19] Su L, Zhang M, Zhang W, Cai C, Hong J. Pretreatment hematologic markers as prognostic factors in patients with nasopharyngeal carcinoma: A systematic review and meta-analysis. Medicine (Baltimore). 2017;96(11):e6364. doi: 10.1097/MD.0000000000006364 28296774 PMC5369929

[20] Zhao W, Li X, Lv L, Sun X, Xue J, Yang P, et al. Systematic review and metanalysis of neutrophil to lymphocyte ratio and prognosis in patients with nasopharyngeal carcinoma. Laryngoscope Investig Otolaryngol. 2023;8(6):1522–31. doi: 10.1002/lio2.1161 38130245 PMC10731536

[21] Takenaka Y, Kitamura T, Oya R, Ashida N, Shimizu K, Takemura K, et al. Prognostic role of neutrophil-lymphocyte ratio in nasopharyngeal carcinoma: A meta-analysis. PLoS One. 2017;12(7):e0181478. doi: 10.1371/journal.pone.0181478 28715474 PMC5513538

[22] Luan C-W, Yang H-Y, Tsai Y-T, Hsieh M-C, Chou H-H, Chen K-S. Prognostic Value of C-Reactive Protein-to-Albumin Ratio in Head and Neck Cancer: A Meta-Analysis. Diagnostics (Basel). 2021;11(3):403. doi: 10.3390/diagnostics11030403 33652976 PMC7996835

[23] Cetintepe T, Cetintepe L, Guc Z, Ertekin I, Karaalp O, Alacacioglu A. LDH-to-Albumin Ratio (LAR) in Solid Tumor Patients with Cytopenia: A Simple Biomarker to Predict Bone Marrow Metastasis. J Clin Med. 2025;14(20):7379. doi: 10.3390/jcm14207379 41156248 PMC12564924

[24] Yin J, Qin Y, Luo Y-K, Feng M, Lang J-Y. Prognostic value of neutrophil-to-lymphocyte ratio for nasopharyngeal carcinoma: A meta-analysis. Medicine (Baltimore). 2017;96(29):e7577. doi: 10.1097/MD.0000000000007577 28723793 PMC5521933

[25] Wang G, Zou X, Weng J, Lan G, Li M, Wei J, et al. Bibliometric analysis reveals the research hotspots and trends of nasopharyngeal carcinoma immunotherapy. Hum Vaccin Immunother. 2024;20(1):2360341. doi: 10.1080/21645515.2024.2360341 39034441 PMC11789730

[26] Yang Y, Hou J, Zhang Y, Sun Y, Fu Y, Lu Q, et al. Radiomics model based on vertebral calcium-suppressed CT images for predicting chemotherapy-induced myelosuppression in nasopharyngeal carcinoma. Front Oncol. 2025;15:1574250. doi: 10.3389/fonc.2025.1574250 40969270 PMC12442038

[27] Schünemann HJ, Mustafa R, Brozek J, Santesso N, Alonso-Coello P, Guyatt G, et al. GRADE Guidelines: 16. GRADE evidence to decision frameworks for tests in clinical practice and public health. J Clin Epidemiol. 2016;76:89–98. doi: 10.1016/j.jclinepi.2016.01.032 26931285

[28] Cho O, Noh OK, Oh Y-T, Chang S-J, Ryu H-S, Lee EJ, et al. Hematological parameters during concurrent chemoradiotherapy as potential prognosticators in patients with stage IIB cervical cancer. Tumour Biol. 2017;39(2):1010428317694306. doi: 10.1177/1010428317694306 28222668

[29] Lee S, Choi Y, Seo M-K, Jang J, Shin N-Y, Ahn K-J, et al. Magnetic Resonance Imaging-Based Radiomics for the Prediction of Progression-Free Survival in Patients with Nasopharyngeal Carcinoma: A Systematic Review and Meta-Analysis. Cancers (Basel). 2022;14(3):653. doi: 10.3390/cancers14030653 35158921 PMC8833585

[30] Mai H-Q, Chen Q-Y, Chen D, Hu C, Yang K, Wen J, et al. Toripalimab Plus Chemotherapy for Recurrent or Metastatic Nasopharyngeal Carcinoma: The JUPITER-02 Randomized Clinical Trial. JAMA. 2023;330(20):1961–70. doi: 10.1001/jama.2023.20181 38015220 PMC10685882

[31] Weidhaas JB, McGreevy KM, Marco N, Sundahl N, Cabanski CR, Spencer C, et al. Germline microRNA-based signatures predict toxicity and response to anti-CTLA-4 therapy. J Transl Med. 2025;23(1):848. doi: 10.1186/s12967-025-06842-3 40721801 PMC12306077

[32] Zhu Y, Liu K, Wang K, Zhu H. Treatment-related adverse events of antibody-drug conjugates in clinical trials: A systematic review and meta-analysis. Cancer. 2023;129(2):283–95. doi: 10.1002/cncr.34507 36408673 PMC10099922

