# Supplementary material for: Prognostic value of the three-lineage cytopenia score in locally advanced nasopharyngeal carcinoma: A retrospective cohort study
Source: PLoS One. 2026 Aug 13;21(8):e0356005. doi: 10.1371/journal.pone.0356005 (PMC13472369; doi:10.1371/journal.pone.0356005)
Supplement: Supplementary file 2 [file pone.0356005.s001.docx]

**Prognostic Value of the Three-Lineage Cytopenia Score in Locally Advanced Nasopharyngeal Carcinoma: A Retrospective Cohort Study**

Yi Li^1^, Zhongyi Li^2^, Junyan He^2^, Yang Li^2^, Yu Duan^2^, Dong Yang^2*^

^1^ Department of Obstetrics and Gynecology, The First Affiliated Hospital, Hengyang Medical School, University of South China, Hengyang421001, Hunan, China.

^2^ Department of Oncology, The First Affiliated Hospital, Hengyang Medical School, University of South China, Hengyang 421001, Hunan, China.

**^*^orresponding author detail**

E-mail: yangdong4168@163.com

**Supplementary Table 1. Distribution of post-treatment myelosuppression indicators in the entire cohort**

| Characteristic | Total Cohort (n=576, %) |
| --- | --- |
|  |  |
| **WBC decline grade** |  |
| None | 179 (31.1) |
| Grade I° | 169 (29.3) |
| Grade II° | 112 (19.4) |
| Grade III-IV° | 116 (20.2) |
| **Hb decline grade** |  |
| None | 126 (21.9) |
| Grade I° | 181 (31.4) |
| Grade II° | 148 (25.7) |
| Grade III-IV° | 121 (21.0) |
| **PLT decline grade** |  |
| None | 353 (61.3) |
| Grade I° | 84 (14.6) |
| Grade II° | 79 (13.7) |
| Grade III-IV° | 60 (10.4) |
| **Three-lineage cytopenia score** |  |
| 0 | 52 (9.0) |
| 1 | 118 (20.5) |
| 2 | 268 (46.5) |
| 3 | 138 (24.0) |

Note: The grading of WBC, Hb, and PLT decline follows the Common Terminology Criteria for Adverse Events (CTCAE). The three-lineage cytopenia score is defined as the number of these three parameters falling below the lower limit of normal.

**Supplementary Table 2. Univariate analysis of post-treatment myelosuppression grades and 5-year survival rates**

| Parameter & Grade | No. of Patients | 5-Year OS (%) | 5-Year LRFS (%) | 5-Year DMFS (%) | Log-rank P |
| --- | --- | --- | --- | --- | --- |
| **Leukopenia** |  |  |  |  | **<0.001** |
| Grade 0 | 179 | 88.8 | 96.6 | 95.5 |  |
| Grade I° | 169 | 77.5 | 91.7 | 80.5 |  |
| Grade II | 112 | 65.2 | 83.9 | 71.4 |  |
| Grade III | 116 | 58.6 | 76.7 | 67.2 |  |
| **Hemoglobin decline** |  |  |  |  | **<0.001** |
| Grade 0 | 126 | 88.9 | 96.0 | 92.1 |  |
| Grade I | 181 | 78.5 | 92.3 | 85.6 |  |
| Grade II | 148 | 67.6 | 87.2 | 76.4 |  |
| Grade III | 121 | 63.6 | 77.7 | 66.9 |  |
| **Thrombocytopenia** |  |  |  |  | **<0.001** |
| Grade 0 | 353 | 80.7 | 92.6 | 87.8 |  |
| Grade I | 84 | 76.2 | 90.5 | 75.0 |  |
| Grade II | 79 | 65.8 | 84.8 | 73.4 |  |
| Grade III | 60 | 50.0 | 68.3 | 56.7 |  |
| **Three-lineage cytopenia score** |  |  |  |  | **<0.001** |
| Score 0 | 52 | 88.5 | 98.1 | 96.2 |  |
| Score 1 | 118 | 89.0 | 94.1 | 97.5 |  |
| Score 2 | 268 | 78.4 | 92.9 | 81.3 |  |
| Score 3 | 138 | 50.7 | 72.5 | 59.4 |  |

Note: Survival rates were estimated using the Kaplan-Meier method. The three-lineage cytopenia score is defined as the sum of the number of lineages (leukocytes, hemoglobin, platelets) below the lower limit of normal.

**Supplementary Table 3. Univariate Cox regression analysis of prognostic factors in NPC patients**

| Characteristics | 5-year OS | | 5-year LRFS | | | 5-year DMFS | | |
| --- | --- | --- | --- | --- | --- | --- | --- | --- |
|  | HR (95%CI) | *P*-Value | HR (95%CI) | *P*-Value | | HR (95%CI) | | *P*-Value |
| **Gender** |  |  |  | |  |  |  | |
| Male | Reference |  | Reference | |  | Reference |  | |
| Female | 0.940(0.640-1.381) | 0.754 | 0.895(0.503-1.592) | | 0.706 | 0.892(0.576-1.381) | 0.609 | |
| **Age (years)** |  |  |  | |  |  |  | |
| <47 | Reference |  | Reference | |  | Reference |  | |
| ≥47 | 0.588(0.422-0.821) | 0.002 | 1.999(1.204-3.318) | | 0.007 | 1.337(0.925-1.932) | 0.122 | |
| **8^th^ T stage** |  | 0.001 |  | | <0.001 |  | 0.573 | |
| T1-2 | Reference |  | Reference | |  | Reference |  | |
| T3 | 1.230(0.739-2.047) | 0.426 | 3.152(0.943-10.531) | | 0.062 | 0.954(0.579-1.573) | 0.853 | |
| T4 | 2.197(1.329-3.632) | 0.002 | 8.796(2.707-28.576) | | <0.001 | 1.184(0.704-1.992) | 0.524 | |
| **8^th^ N stage** |  | <0.001 |  | | 0.114 |  | <0.001 | |
| N0-N1 | Reference |  | Reference | |  | Reference |  | |
| N2 | 1.421(0.886-2.280) | 0.145 | 1.941(0.947-3.977) | | 0.070 | 1.930(1.008-3.696) | 0.047 | |
| N3 | 3.314(1.988-5.524) | <0.001 | 2.385(1.019-5.585) | | 0.045 | 7.790(4.058-14.957) | <0.001 | |
| **Clinical stage** |  |  |  | |  |  |  | |
| III | Reference |  | Reference | |  | Reference |  | |
| IVa | 2.487(1.769-3.496) | <0.001 | 2.704(1.620-4.515) | | <0.001 | 2.617(1.778-3.852) | <0.001 | |
| **ECOG** |  |  |  | |  |  |  | |
| 0 | Reference |  | Reference | |  | Reference |  | |
| 1 | 2.626(1.826-3.775) | <0.001* | 3.006(1.727-5.230) | | <0.001 | 2.594(1.729-3.892) | <0.001 | |
| **Histology type** |  |  |  | |  |  |  | |
| NKUC | Reference |  | Reference | |  | Reference |  | |
| others | 2.219(1.038-4.747) | 0.040 | 4.300(1.855-9.966) | | 0.001 | 1.528(0.563-4.145) | 0.405 | |
| **Drinking history** |  |  |  | |  |  |  | |
| No | Reference |  | Reference | |  | Reference |  | |
| Yes | 1.155(0.740-1.804) | 0.525 | 0.848(0.405-1.778) | | 0.663 | 1.501(0.943-2.390) | 0.087 | |
| **Smoking history** |  |  |  | |  |  |  | |
| No | Reference |  | Reference | |  | Reference |  | |
| Yes | 0.940(0.666-1.328) | 0.727 | 1.088 (0.658-1.796) | | 0.743 | 1.111(0.760-1.624) | 0.587 | |
| **Albumin** |  |  |  | |  |  |  | |
| <43.4 g/L | Reference |  | Reference | |  | Reference |  | |
| ≥43.4 g/L | 0.622(0.448-0.864) | 0.005 | 0.656(0.403-1.068) | | 0.090 | 0.825(0.572-1.90) | 0.304 | |
| **CRP** |  |  |  | |  |  |  | |
| Normal | Reference |  | Reference | |  | Reference |  | |
| Elevated | 0.935(0.634-1.378) | 0.733 | 0.947(0.532-1.687) | | 0.854 | 0.742 (0.466-1.181) | 0.208 | |
| **Pre-treatment EBV-DNA** |  |  |  | |  |  |  | |
| ＜400copies/mL | Reference |  | Reference | |  | Reference |  | |
| ≥400copies/mL | 1.585(1.135-2.215) | 0.007 | 2.128(1.267-3.576) | | 0.004 | 1.611 (1.105-2.349) | 0.013 | |
| **Post-treatment EBV-DNA** |  |  |  | |  |  |  | |
| ＜400copies/mL | Reference |  | Reference | |  | Reference |  | |
| ≥400copies/mL | 2.293(1.651-3.185) | <0.001 | 3.194(1.964-5.195) | | <0.001 | 2.302 (1.588-3.336) | <0.001 | |
| **Maintenance therapy** |  |  |  | |  |  |  | |
| No | Reference |  | Reference | |  | Reference |  | |
| Yes | 0.924(0.668-1.279) | 0.635 | 1.340(0.822-2.183) | | 0.241 | 0.694(0.479-1.006) | 0.054 | |
| **Three-lineage cytopenia score** |  | <0.001 |  | | <0.001 |  | <0.001 | |
| 0 | Reference |  | Reference | |  | Reference |  | |
| 1 | 1.185(0.449-3.130) | 0.802 | 3.719(0.456-30.311) | | 0.220 | 1.063(0.194-5.818) | 0.944 | |
| 2 | 2.324(1.002-5.391) | 0.050 | 4.654(0.624-34.720) | | 0.134 | 6.285(1.530-25.824) | 0.011 | |
| 3 | 7.026(3.027-16..309) | <0.001 | 22.723(3.098-166.688) | | 0.002 | 17.891(4.338-73.780) | <0.001 | |

Note: Abbreviations: HR, hazard ratio; CI, confidence interval; NKUC, non-keratinizing undifferentiated carcinoma; CRP, C-reactive protein; EBV, Epstein-Barr virus.

**Supplementary Table 4. Subgroup analysis of the three-lineage cytopenia score for overall survival**

| Subgroup | Category | No. of Patients | HR (95% CI) | P-value |
| --- | --- | --- | --- | --- |
| **Gender** |  |  |  |  |
|  | Male | 437 | 2.533 (1.940–3.306) | <0.001 |
|  | Female | 139 | 1.777 (1.088–2.904) | 0.022 |
| **Age** |  |  |  |  |
|  | <47 years | 283 | 2.095 (1.460–3.006) | <0.001 |
|  | ≥47 years | 293 | 2.426 (1.793–3.282) | <0.001 |
| **8^th^ T stage** |  |  |  |  |
|  | T1–2 | 104 | 2.688 (1.354–5.334) | 0.005 |
|  | T3–4 | 472 | 2.340 (1.830–2.993) | <0.001 |
| **8^th^ N stage** |  |  |  |  |
|  | N0–1 | 132 | 2.782 (1.487–5.205) | 0.001 |
|  | N2–3 | 444 | 2.251 (1.755–2.887) | <0.001 |
| **ECOG** |  |  |  |  |
|  | 0 | 276 | 1.933 (1.265–2.955) | 0.002 |
|  | 1 | 300 | 2.488 (1.889–3.279) | <0.001 |
| **Albumin** |  |  |  |  |
|  | <43.4 g/L | 275 | 2.070 (1.571–2.728) | <0.001 |
|  | ≥43.4 g/L | 301 | 2.922 (1.959–4.359) | <0.001 |
| **Maintenance therapy** |  |  |  |  |
|  | No | 287 | 2.321 (1.701–3.168) | <0.001 |
|  | Yes | 289 | 2.335 (1.655–3.295) | <0.001 |
| **Post-treatment EBV-DNA** |  |  |  |  |
|  | <400 copies/mL | 411 | 2.469 (1.805–3.378) | <0.001 |
|  | ≥400 copies/mL | 165 | 1.982 (1.409–2.788) | <0.001 |
| **Overall** | All patients | 576 | **2.341 (1.858–2.951)** | **<0.001** |

Note: HR represents the hazard ratio per 1-point increase in the three-lineage cytopenia score, derived from univariate Cox regression models with the score as the sole covariate within each subgroup. CI, confidence interval.

**Supplementary Table 5. Landmark sensitivity analysis (7 months after treatment initiation) for overall survival (OS), locoregional relapse‑free survival (LRFS), and distant metastasis‑free survival (DMFS) by cytopenia grade and three‑lineage cytopenia score**

| Endpoint | Group | Comparison | Log‑rank P (original) | Log‑rank P (landmark) | Interpretation |
| --- | --- | --- | --- | --- | --- |
| OS | Leukopenia | 0 vs I° | 0.003 | 0.001 | significant |
|  |  | 0 vs II | <0.001 | <0.001 | significant |
|  |  | 0 vs III | <0.001 | <0.001 | significant |
|  |  | I° vs II | 0.015 | 0.044 | significant |
|  |  | I° vs III | 0.001 | 0.002 | significant |
|  |  | II vs III | 0.448 | 0.364 | non‑significant |
|  | Hemoglobin | 0 vs I | 0.019 | 0.049 | significant |
|  |  | 0 vs II | <0.001 | 0.023 | significant |
|  |  | 0 vs III | <0.001 | <0.001 | significant |
|  |  | I vs II | 0.040 | 0.710 | non‑significant |
|  |  | I vs III | 0.002 | 0.006 | significant |
|  |  | II vs III | 0.197 | 0.025 | significant |
|  | Thrombocytopenia | 0 vs I | 0.172 | 0.518 | non‑significant |
|  |  | 0 vs II | <0.001 | 0.017 | significant |
|  |  | 0 vs III | <0.001 | <0.001 | significant |
|  |  | I vs II | 0.106 | 0.026 | significant |
|  |  | I vs III | 0.001 | 0.001 | significant |
|  |  | II vs III | 0.053 | 0.188 | non‑significant |
|  | Three‑lineage score | 0 vs 1 | 0.580 | 0.836 | non‑significant |
|  |  | 0 vs 2 | 0.016 | 0.148 | non‑significant |
|  |  | 0 vs 3 | <0.001 | <0.001 | significant |
|  |  | 1 vs 2 | 0.022 | 0.064 | borderline |
|  |  | 1 vs 3 | <0.001 | <0.001 | significant |
|  |  | 2 vs 3 | <0.001 | <0.001 | significant |
| LRFS | Leukopenia | 0 vs I° | 0.039 | 0.026 | significant |
|  |  | 0 vs II | <0.001 | 0.001 | significant |
|  |  | 0 vs III | <0.001 | <0.001 | significant |
|  |  | I° vs II | 0.028 | 0.223 | non‑significant |
|  |  | I° vs III | <0.001 | 0.001 | significant |
|  |  | II vs III | 0.219 | 0.058 | borderline |
|  | Hemoglobin | 0 vs I | 0.175 | 0.152 | non‑significant |
|  |  | 0 vs II | 0.009 | 0.455 | non‑significant |
|  |  | 0 vs III | 0.001 | <0.001 | significant |
|  |  | I vs II | 0.107 | 0.450 | non‑significant |
|  |  | I vs III | <0.001 | 0.009 | significant |
|  |  | II vs III | 0.018 | 0.002 | significant |
|  | Thrombocytopenia | 0 vs I | 0.381 | 0.747 | non‑significant |
|  |  | 0 vs II | 0.007 | 0.897 | non‑significant |
|  |  | 0 vs III | <0.001 | 0.031 | significant |
|  |  | I vs II | 0.234 | 0.899 | non‑significant |
|  |  | I vs III | <0.001 | 0.074 | borderline |
|  |  | II vs III | 0.019 | 0.115 | non‑significant |
|  | Three‑lineage score | 0 vs 1 | 0.295 | 0.249 | non‑significant |
|  |  | 0 vs 2 | 0.147 | 0.109 | non‑significant |
|  |  | 0 vs 3 | 0.004 | 0.012 | significant |
|  |  | 1 vs 2 | 0.639 | 0.316 | non‑significant |
|  |  | 1 vs 3 | <0.001 | 0.008 | significant |
|  |  | 2 vs 3 | <0.001 | 0.015 | significant |
| DMFS | Leukopenia | 0 vs I° | <0.001 | <0.001 | significant |
|  |  | 0 vs II | <0.001 | <0.001 | significant |
|  |  | 0 vs III | <0.001 | <0.001 | significant |
|  |  | I° vs II | 0.039 | 0.427 | non‑significant |
|  |  | I° vs III | 0.007 | 0.020 | significant |
|  |  | II vs III | 0.612 | 0.198 | non‑significant |
|  | Hemoglobin | 0 vs I | 0.092 | 0.527 | non‑significant |
|  |  | 0 vs II | 0.001 | 0.089 | borderline |
|  |  | 0 vs III | <0.001 | 0.003 | significant |
|  |  | I vs II | 0.033 | 0.223 | non‑significant |
|  |  | I vs III | <0.001 | 0.009 | significant |
|  |  | II vs III | 0.028 | 0.158 | non‑significant |
|  | Thrombocytopenia | 0 vs I | 0.001 | 0.182 | non‑significant |
|  |  | 0 vs II | <0.001 | 0.017 | significant |
|  |  | 0 vs III | <0.001 | <0.001 | significant |
|  |  | I vs II | 0.660 | 0.444 | non‑significant |
|  |  | I vs III | 0.010 | 0.020 | significant |
|  |  | II vs III | 0.047 | 0.111 | non‑significant |
|  | Three‑lineage score | 0 vs 1 | 0.724 | 0.652 | non‑significant |
|  |  | 0 vs 2 | 0.004 | 0.208 | non‑significant |
|  |  | 0 vs 3 | <0.001 | 0.001 | significant |
|  |  | 1 vs 2 | <0.001 | 0.023 | significant |
|  |  | 1 vs 3 | <0.001 | <0.001 | significant |
|  |  | 2 vs 3 | <0.001 | <0.001 | significant |

Note: Landmark time point was set at 7 months after treatment initiation. Patients with events or censoring before this landmark were excluded. “Borderline” indicates 0.05 ≤ P < 0.10.

**Supplementary Table 6. Sensitivity analysis for OS, LRFS, and DMFS adjusting for baseline blood counts (three‑lineage cytopenia score as a continuous variable)**

| Endpoint | HR per 1‑point increase (95% CI) | P‑value | Events (n) |
| --- | --- | --- | --- |
| OS | 2.182 (1.741–2.734) | <0.001 | 145 |
| LRFS | 2.994 (2.053–4.368) | <0.001 | 65 |
| DMFS | 3.052 (2.270–4.103) | <0.001 | 111 |

Note: All models adjusted for age, T/N stage, ECOG, histology, albumin, pre‑/post‑treatment EBV‑DNA, drinking history, maintenance therapy (as appropriate), and baseline white blood cell, hemoglobin, and platelet counts. Full results for all covariates are available from the corresponding author upon request.
