# Supplementary material for: Prognostic value of the three-lineage cytopenia score in locally advanced nasopharyngeal carcinoma: A retrospective cohort study
Source: PLoS One. 2026 Aug 13;21(8):e0356005. doi: 10.1371/journal.pone.0356005 (PMC13472369; doi:10.1371/journal.pone.0356005)
Supplement: Supplementary file 3 [file pone.0356005.s004.docx]

**Response to Reviewers**

**Manuscript ID:** PONE-D-26-14203

**Title:** Prognostic Value of the Three-Lineage Cytopenia Score in Locally Advanced Nasopharyngeal Carcinoma: A Retrospective Cohort Study

Dear Editor and Reviewers,

We thank the Editor and the Reviewers for their thoughtful comments and constructive suggestions. We have carefully addressed all concerns and have revised the manuscript accordingly. All changes have been highlighted in yellow in the revised manuscript. Below we provide a point‑by‑point response to each comment.

**Response to Reviewer #1**

*General comment: The manuscript addresses a clinically relevant question, but the core analytical framework contains major methodological problems.*

**Comment 1 (Time‑dependent bias / guarantee‑time bias):**

The cytopenia score was derived from nadir values during treatment, while survival was measured from treatment initiation. This creates a risk of guarantee‑time bias. A landmark analysis or time‑dependent Cox model would be more appropriate.

**Our response:** We agree with the reviewer. To address guarantee‑time bias, we performed a landmark sensitivity analysis. The landmark time point was set at 7 months after treatment initiation (based on median treatment duration of approximately 4 months plus 90 days). Patients who had an event or were censored before this landmark were excluded, and survival analyses were repeated. The results of this landmark analysis are presented in **Supplementary Table 5**. The landmark analysis showed results consistent with the primary analysis, confirming the robustness of our findings. We have added a description of this analysis in the Methods section (highlighted in yellow) and cited the supplementary table.

**Comment 2 (Residual confounding – baseline blood counts):**

The score may simply reflect pre‑existing bone marrow reserve (baseline WBC, hemoglobin, platelets). The multivariable model did not adjust for baseline values.

**Our response:** We appreciate this important concern. We performed a sensitivity analysis by additionally adjusting for baseline white blood cell, hemoglobin, and platelet counts in the multivariate Cox models for all three endpoints. The results (presented in **Supplementary Table 6**) show that the three‑lineage cytopenia score remained strongly and independently associated with OS, LRFS, and DMFS (as a continuous variable). These findings indicate that the prognostic value of the score is not simply a reflection of baseline hematological status. The sensitivity analysis has been described in the Methods (highlighted in yellow) and the results are cited.

**Comment 3 (Heterogeneity of treatment):**

The therapeutic context is highly heterogeneous (different chemotherapy regimens, dose modifications, G‑CSF use, etc.), and the manuscript provides insufficient adjustment for these factors.

**Our response:** We acknowledge this limitation. The retrospective, single‑center design does not allow us to fully capture all treatment modifications and supportive care interventions. We have added a statement in the Discussion (highlighted in yellow) acknowledging this as a limitation and calling for future multicenter studies with more detailed treatment data.

**Comment 4 (Overstated dose‑response relationship):**

The data do not support a clear dose‑response relationship; the association is driven mainly by the score 3 subgroup. Pairwise comparisons between scores 0, 1, and 2 are often non‑significant.

**Our response:** We agree with the reviewer. We have revised the wording throughout the manuscript to avoid claiming a “clear dose‑response relationship” or “progressive stepwise decrease”. Instead, we now state that the association is most pronounced for patients with a score of 3 (involvement of all three lineages), and that differences between scores 0, 1, and 2 are either non‑significant or inconsistent across endpoints. These changes have been highlighted in yellow in the Results section (Three‑lineage cytopenia score and prognosis).

**Comment 5 (Poor prognostic model performance and AUC <0.5 for TNM model):**

The model achieved an AUC of only 0.593, and the TNM‑only model had an AUC below 0.5, raising concerns about model specification.

**Our response:** We agree that the discriminatory ability of our model is modest (AUC=0.593). We have revised the Results accordingly, stating that the model shows “modest discriminatory ability”. Regarding the TNM‑only model, we found that TNM staging alone performed poorly in our cohort (AUC <0.5), possibly due to sample characteristics. Therefore, we decided to remove the formal comparison with the TNM model and only present the ROC curve for our new model. The revised Figure 3B now shows only the ROC curve for the model incorporating the three‑lineage cytopenia score, and the corresponding text has been modified (highlighted in yellow). We also added a sentence in the Discussion acknowledging that the AUC leaves room for improvement.

**Comment 6 (Unstable effect estimates, wide confidence intervals):**

The hazard ratios for LRFS and DMFS in the score 3 group have extremely wide confidence intervals (e.g., 2.567–149.582).

**Our response:** We agree that the wide confidence intervals reflect the limited number of events, especially for LRFS (65 events). We have added a note in the Results (pairwise comparisons) and in the Discussion (limitations) to caution against overinterpretation of these estimates. These additions are highlighted in yellow.

**Response to Reviewer #2**

**Comment 1 (Timing of laboratory data collection):**

The authors should indicate the exact time points when laboratory data were collected after treatment.

**Our response:** We have added the following statement to the Methods section (highlighted in yellow): “Complete blood counts were measured at least weekly during radiotherapy and chemotherapy cycles. The nadir values were recorded as the lowest counts during the entire treatment period.”

**Comment 2 (Use of absolute WBC count instead of differential counts):**

The authors should provide a justification for using absolute white blood cell count rather than differential counts.

**Our response:** We have added a justification in the Methods (highlighted in yellow): “The total white blood cell count was chosen because it reflects overall leukocyte suppression, which is clinically relevant for myelosuppression grading. Differential counts were not analyzed to avoid multiple comparisons and because the three‑lineage cytopenia score is intended as a simple, practical tool using routine complete blood count parameters.”

**Response to Reviewer #3**

**Comment 1 (Provide cut‑off points for single‑lineage cytopenias):**

The authors should provide the cut‑off values used for grading leukopenia, anemia, and thrombocytopenia.

**Our response:** We have added the CTCAE version 5.0 cut‑off points in the Methods section (highlighted in yellow). These include the exact numerical thresholds for grades 1–4 for each lineage.

**Comment 2 (Report percentages of patients who died, had recurrence, and developed distant metastasis):**

These percentages are already present in the Results (first paragraph of Survival Outcomes).

**Our response:** We confirm that the percentages are already reported (e.g., “145 patients died (25.2%)”). No additional change was needed.

**Comment 3 (Improve reporting of results by citing specific figure panels):**

The authors should cite specific figure panels (e.g., Figure 1A–C) rather than giving only general statements.

**Our response:** We have revised the Results section to cite specific figure panels throughout. For example, “Figure 1A–C” instead of “Figure 1”. All changes are highlighted in yellow.

**Additional Revisions (Editor’s requests)**

**1. Style requirements:** The manuscript has been formatted according to PLOS ONE’s style templates.

**2. Ethics statement:** The ethics statement appears only in the Methods section (as required).

**3. Data availability statement:** We have updated the Data Availability Statement in the submission system as follows: “Due to ethical restrictions… data are available upon reasonable request from the corresponding author with approval from the ethics committee.” The statement is also included in the manuscript.

**4. Financial disclosure role of funders:** We have added the following sentence to the cover letter: “The funders had no role in study design, data collection and analysis, decision to publish, or preparation of the manuscript.”

**5. Supporting Information captions:** Captions for all supplementary tables have been added at the end of the manuscript.

**6. Landmark and baseline‑adjusted sensitivity analyses:** These have been added as Supplementary Tables 5 and 6, as described above.

**7. Figure 3B:** We have removed the TNM‑only ROC curve and now present only the curve for our model, with updated text and legend.

We believe that the revised manuscript has been substantially improved and addresses all concerns raised by the reviewers. We thank the Editor and Reviewers for their time and valuable input. We look forward to your favorable decision.

Sincerely,

Dong Yang, MD
Corresponding author
