# Supplementary material for: Prognostic value of the three-lineage cytopenia score in locally advanced nasopharyngeal carcinoma: A retrospective cohort study
Source: PLoS One. 2026 Aug 13;21(8):e0356005. doi: 10.1371/journal.pone.0356005 (PMC13472369; doi:10.1371/journal.pone.0356005)
Supplement: Supplementary file 4 [file pone.0356005.s005.docx]

**Response to Reviewers**

**Manuscript ID:** PONE-D-26-14203R1

**Title:** Prognostic Value of the Three-Lineage Cytopenia Score in Locally Advanced Nasopharyngeal Carcinoma: A Retrospective Cohort Study

Dear Editor and Reviewers,

We sincerely thank the Academic Editor and the Reviewers for their thoughtful and constructive comments on our revised manuscript. We are grateful for the positive feedback and the recognition that our previous revisions have substantially improved the manuscript. We have now carefully addressed all remaining concerns raised in this round of review. All changes have been highlighted in yellow in the revised manuscript. Below we provide a point-by-point response to each comment.

**Response to Reviewer #1**

*General comment: The authors have addressed the major methodological concerns, but several issues remain.*

**Comment 1 (Inconsistency regarding ROC analysis and TNM-only model):**

The response letter states that the formal comparison with the TNM-only model was removed because the TNM-only model showed an AUC below 0.5. However, the Abstract and parts of the manuscript still contain statements suggesting improved discrimination versus TNM staging alone. Given the modest AUC of 0.593 and the problematic performance of the TNM-only model, claims regarding incremental predictive value should be further toned down or removed.

**Our response:** We thank the reviewer for this careful observation. We have revised the Abstract to remove any suggestion of comparison with the TNM-only model. The Abstract now states that the model yielded an AUC of 0.593 (95% CI: 0.533–0.652, P=0.001), indicating modest discriminatory ability, without claiming superiority over TNM staging. Similarly, in the Results section (3.4.1), we have modified the concluding sentence to state that the score “may provide additional prognostic information,” while acknowledging that the modest AUC indicates further refinement is needed. In the Discussion, we have changed “significantly improved” to “showed modest improvement.” All changes are highlighted in yellow.

**Comment 2 (Overstatement of clinical utility):**

Although the three-lineage cytopenia score appears to be associated with survival outcomes, the discriminatory performance remains modest, and some estimates have wide confidence intervals. Statements such as “strong predictive value,” “improve prognostic accuracy,” or “guide personalized treatment” should be revised to more cautious wording.

**Our response:** We agree with the reviewer. We have revised the Abstract, Discussion, and Conclusion throughout to adopt more cautious wording. Specifically:

- In the Abstract, “strong predictive value” has been replaced with “promising predictive value,” and “improve prognostic accuracy and guide personalized treatment” has been changed to “serve as a useful adjunct to traditional staging for risk stratification.”
- In the Discussion (clinical implications paragraph), we have removed “effective” and changed “should consider” to “may consider,” adding that “prospective validation is needed before clinical implementation.”
- In the Conclusion, “strong predictive value” has been changed to “promising predictive value,” “enhances prognostic accuracy” to “may provide additional prognostic information,” and “valuable tool for personalized treatment” to “potential tool for risk stratification.”

All changes are highlighted in yellow.

**Comment 3 (Reporting of sensitivity analyses):**

The addition of the landmark analysis and baseline blood count-adjusted analysis is appreciated. However, these analyses should be reported more transparently, including the number of patients excluded, the number of patients included, events, HRs, 95% CIs, and P values.

**Our response:** We have added a new paragraph in the Results section (Survival Outcomes) reporting the landmark analysis results in detail. Specifically, we now state that 36 patients (6.3%) were excluded from the OS analysis, leaving 540 patients with 82 events, and that the three-lineage cytopenia score (as a continuous variable) remained significantly associated with OS (HR=2.182 per 1‑point increase; 95% CI: 1.741–2.734; P<0.001), with consistent results for LRFS and DMFS. Full details are also provided in Supplementary Table 5. This change is highlighted in yellow.

**Comment 4 (Treatment heterogeneity and supportive care):**

Treatment heterogeneity remains an important issue because cytopenia may be influenced by chemotherapy regimen, treatment intensity, dose reduction, G‑CSF use, transfusion, and other supportive care. These variables should be summarized or, if unavailable, the limitation should be described more explicitly.

**Our response:** We acknowledge that detailed information on G‑CSF use, transfusion support, and chemotherapy dose modifications was not uniformly available in this retrospective dataset. We have added a clear statement to this effect in the Limitations paragraph of the Discussion (highlighted in yellow). We also note that these unmeasured factors may have influenced the observed associations and emphasize the need for future prospective studies with comprehensive treatment and supportive care documentation.

**Comment 5 (Consistency between the response letter and revised manuscript):**

Please carefully check the manuscript to ensure that all revisions described in the response letter are fully reflected in the Abstract, Methods, Results, Discussion, figure legends, and supplementary material.

**Our response:** We have carefully reviewed the entire manuscript and confirmed that all revisions described in our previous response letter have been incorporated. We have also verified that the Abstract, Methods, Results, Discussion, figure legends, and supplementary materials are internally consistent and accurately reflect the changes made.

**Response to Reviewer #2**

*General comment: The manuscript has been substantially improved.*

**Our response:** We sincerely thank the reviewer for the positive feedback and for acknowledging the improvements made to the manuscript. We are grateful for the reviewer’s time and thoughtful evaluation.

**Response to Reviewer #3**

*General comment: The authors should integrate the results of landmark analysis in the main results of the manuscript, rather than only citing them in the methods and discussion sections and relegating them to supplementary materials.*

**Our response:** We agree with the reviewer. We have now integrated the landmark analysis results into the main Results section. A new paragraph has been added in the Survival Outcomes section (highlighted in yellow), which reports the key findings of the landmark sensitivity analysis, including the number of patients excluded, the number of patients included and events, and the HRs with 95% CIs and P values for all three endpoints. The detailed results remain available in Supplementary Table 5 for readers who wish to examine the complete data.

**Additional Revisions**

In addition to the reviewer-specific changes, we have made the following adjustments:

- In the Discussion (first paragraph, point 3), we changed “significantly improved” to “showed modest improvement” to better reflect the modest AUC.
- In the Results section (3.4.1), we revised the concluding sentence to state that the score “may provide additional prognostic information” while acknowledging the modest AUC indicates further refinement is needed.
- The Abstract has been revised to remove any comparison with the TNM-only model and to adopt more cautious wording regarding predictive value and clinical utility.

We believe that the revised manuscript has been further strengthened and now addresses all remaining concerns. We are grateful for the reviewers’ time, expertise, and constructive suggestions, which have significantly improved our work. We look forward to your favorable decision.

Sincerely,

Dong Yang, MD

Corresponding author
