# Supplementary material for: Prognostic value of the three-lineage cytopenia score in locally advanced nasopharyngeal carcinoma: A retrospective cohort study
Source: PLoS One. 2026 Aug 13;21(8):e0356005. doi: 10.1371/journal.pone.0356005 (PMC13472369; doi:10.1371/journal.pone.0356005)
Supplement: Peer review history

# Peer review: Decision Letter 0

**DOI:** 10.1371/journal.pone.0356005.r001

**Authors:** Satyajeet Rath

21 May 2026

Dear Dr. yang,

Thank you for submitting your manuscript to PLOS ONE. After careful consideration, we feel that it has merit but does not fully meet PLOS ONE’s publication criteria as it currently stands. Therefore, we invite you to submit a revised version of the manuscript that addresses the points raised during the review process.

==============================

**ACADEMIC EDITOR: Major revision**

Please submit your revised manuscript by Jul 05 2026 11:59PM. If you will need more time than this to complete your revisions, please reply to this message or contact the journal office at plosone@plos.org. When you're ready to submit your revision, log on to https://www.editorialmanager.com/pone/ and select the 'Submissions Needing Revision' folder to locate your manuscript file.

- A letter that responds to each point raised by the academic editor and reviewer(s). You should upload this letter as a separate file labeled 'Response to Reviewers'.
- A marked-up copy of your manuscript that highlights changes made to the original version. You should upload this as a separate file labeled 'Revised Manuscript with Track Changes'.
- An unmarked version of your revised paper without tracked changes. You should upload this as a separate file labeled 'Manuscript'.

If you would like to make changes to your financial disclosure, please include your updated statement in your cover letter. Guidelines for resubmitting your figure files are available below the reviewer comments at the end of this letter.

If applicable, we recommend that you deposit your laboratory protocols in protocols.io to enhance the reproducibility of your results. Protocols.io assigns your protocol its own identifier (DOI) so that it can be cited independently in the future. For instructions see: https://journals.plos.org/plosone/s/submission-guidelines#loc-laboratory-protocols. Additionally, PLOS ONE offers an option for publishing peer-reviewed Lab Protocol articles, which describe protocols hosted on protocols.io. Read more information on sharing protocols at https://plos.org/protocols?utm_medium=editorial-email&utm_source=authorletters&utm_campaign=protocols.

As the corresponding author, your ORCID iD is verified in the submission system and will appear in the published article. PLOS supports the use of ORCID, and we encourage all coauthors to register for an ORCID iD and use it as well. Please encourage your coauthors to verify their ORCID iD within the submission system before final acceptance, as unverified ORCID iDs will not appear in the published article. *Only*  the individual author can complete the verification step; PLOS staff *cannot*  verify ORCID iDs on behalf of authors.

We look forward to receiving your revised manuscript.

Kind regards,

Satyajeet Rath

Academic Editor

PLOS One

Journal Requirements:

When submitting your revision, we need you to address these additional requirements.

1. Please ensure that your manuscript meets PLOS ONE's style requirements, including those for file naming. The PLOS ONE style templates can be found at

https://journals.plos.org/plosone/s/file?id=wjVg/PLOSOne_formatting_sample_main_body.pdf and https://journals.plos.org/plosone/s/file?id=ba62/PLOSOne_formatting_sample_title_authors_affiliations.pdf

2. Your ethics statement should only appear in the Methods section of your manuscript. If your ethics statement is written in any section besides the Methods, please delete it from any other section.

3. We note that you have indicated that there are restrictions to data sharing for this study. For studies involving human research participant data or other sensitive data, we encourage authors to share de-identified or anonymized data. However, when data cannot be publicly shared for ethical reasons, we allow authors to make their data sets available upon request. For information on unacceptable data access restrictions, please see http://journals.plos.org/plosone/s/data-availability#loc-unacceptable-data-access-restrictions.

Before we proceed with your manuscript, please address the following prompts:

a) If there are ethical or legal restrictions on sharing a de-identified data set, please explain them in detail (e.g., data contain potentially identifying or sensitive patient information, data are owned by a third-party organization, etc.) and who has imposed them (e.g., a Research Ethics Committee or Institutional Review Board, etc.). Please also provide contact information for a data access committee, ethics committee, or other institutional body to which data requests may be sent.

b) If there are no restrictions, please upload the minimal anonymized data set necessary to replicate your study findings to a stable, public repository and provide us with the relevant URLs, DOIs, or accession numbers. Please see http://www.bmj.com/content/340/bmj.c181.long for guidelines on how to de-identify and prepare clinical data for publication. For a list of recommended repositories, please see https://journals.plos.org/plosone/s/recommended-repositories. You also have the option of uploading the data as Supporting Information files, but we would recommend depositing data directly to a data repository if possible.

Please update your Data Availability statement in the submission form accordingly.

4. Thank you for stating the following financial disclosure:

This work was supported by the Natural Science Foundation of Hunan Province [Grant Number 2026JJ80198] and the Doctoral Startup Fund of the First Affiliated Hospital of University of South China.

Please state what role the funders took in the study. If the funders had no role, please state: "The funders had no role in study design, data collection and analysis, decision to publish, or preparation of the manuscript."

If this statement is not correct you must amend it as needed.

Please include this amended Role of Funder statement in your cover letter; we will change the online submission form on your behalf.

5. Please include captions for your Supporting Information files at the end of your manuscript, and update any in-text citations to match accordingly. Please see our Supporting Information guidelines for more information: http://journals.plos.org/plosone/s/supporting-information.

6. If the reviewer comments include a recommendation to cite specific previously published works, please review and evaluate these publications to determine whether they are relevant and should be cited. There is no requirement to cite these works unless the editor has indicated otherwise.

Additional Editor Comments:

Major revision

[Note: HTML markup is below. Please do not edit.]

Reviewers' comments:

Reviewer's Responses to Questions

**Comments to the Author**

1. Is the manuscript technically sound, and do the data support the conclusions?

Reviewer #1: No

Reviewer #2: Yes

Reviewer #3: Yes

**********

2. Has the statistical analysis been performed appropriately and rigorously?

Reviewer #1: No

Reviewer #2: Yes

Reviewer #3: Yes

**********

3. Have the authors made all data underlying the findings in their manuscript fully available?

The PLOS Data policy

Reviewer #1: Yes

Reviewer #2: No

Reviewer #3: Yes

**********

4. Is the manuscript presented in an intelligible fashion and written in standard English?

Reviewer #1: Yes

Reviewer #2: Yes

Reviewer #3: Yes

**********

Reviewer #1: This manuscript addresses a clinically relevant question, namely whether treatment-related multilineage cytopenia has prognostic value in patients with locally advanced nasopharyngeal carcinoma. The cohort size is relatively large, and the authors propose a simple and potentially practical score based on leukocyte, hemoglobin, and platelet suppression. However, despite the apparent clinical interest of the topic, I do not believe that the current manuscript is suitable for publication in its present form because the core analytical framework contains major methodological problems that substantially limit the validity and interpretability of the conclusions.

My primary concern is the temporal structure of the analysis. The three-lineage cytopenia score was constructed from nadir blood counts recorded during treatment, and the manuscript also refers to this as a “post-treatment” prognostic factor. However, overall survival, locoregional relapse-free survival, and distant metastasis-free survival were all calculated from treatment initiation, while the inclusion criteria required patients to have completed the planned treatment course and excluded those who failed to complete treatment. This creates a major risk of time-dependent bias or guarantee-time bias, because the exposure variable is only fully observable after treatment has progressed, whereas the survival clock starts earlier. As currently analyzed, the score cannot be cleanly interpreted as a baseline-independent prognostic factor. A landmark analysis or a time-dependent Cox model would have been far more appropriate.

A second major issue is residual confounding and construct ambiguity. The score is presented as a treatment-related toxicity marker, yet it may simply reflect poorer baseline marrow reserve, frailty, nutritional status, or systemic inflammation. The manuscript states that pre-treatment clinical and laboratory variables were collected, including albumin, CRP, and blood counts, but the multivariable model did not adjust for baseline WBC, hemoglobin, or platelet values themselves. In fact, the Discussion explicitly acknowledges that myelosuppression may act as a “signal” of poor baseline health status, and the authors note differences in pre-treatment CRP across groups. Therefore, the current model does not convincingly separate treatment-emergent toxicity from pre-existing patient vulnerability.

Third, the therapeutic context is highly heterogeneous, but this heterogeneity is not handled adequately. Patients received different concurrent cisplatin schedules, multiple induction regimens, platinum substitutions, and even maintenance therapy that included either capecitabine alone or capecitabine combined with PD-1/PD-L1 inhibitors. These differences are highly relevant because treatment intensity and regimen selection are directly linked to hematologic toxicity. Yet the manuscript provides no sufficiently detailed adjustment for dose reductions, delays, cumulative dose intensity, G-CSF use, transfusion support, or other toxicity-management interventions. Without such information, it is difficult to know whether the score reflects biology, treatment intensity, treatment tolerance, or supportive-care patterns.

Fourth, the data do not fully support the authors’ repeated claim of a clear dose-response relationship. In the reported Kaplan-Meier results, the 5-year OS for score 1 was actually slightly higher than for score 0 (89.0% vs 88.5%), and the 5-year DMFS was also slightly higher in score 1 than in score 0 (97.5% vs 96.2%). Several pairwise comparisons involving scores 0, 1, and 2 were not significant. This suggests that the observed association is driven mainly by the score 3 subgroup rather than by a robust monotonic gradient across the full score range. The manuscript therefore overstates the strength and consistency of the dose-response pattern.

Fifth, the prognostic model performance is not convincing. The model incorporating the cytopenia score achieved an AUC of only 0.593, which indicates poor discrimination. More concerningly, the comparator TNM-only model reportedly had an AUC of 0.372, which is below 0.5 and raises questions about model specification, outcome coding, or ROC implementation. In addition, the reported 5-year OS was 78.6% in the low-risk group and 82.8% in the intermediate-risk group, which is not directionally coherent for a clinically useful risk stratification model. These findings substantially weaken the claim that the new score meaningfully improves prognostic prediction.

Sixth, several effect estimates appear unstable. For example, the hazard ratio for LRFS in score 3 was 19.596 with an extremely wide 95% confidence interval of 2.567–149.582, and similarly wide intervals were reported for DMFS. Such imprecision suggests limited robustness and possible overfitting relative to the number of events. This is particularly relevant because the manuscript advances strong clinical conclusions regarding distant metastasis prediction and personalized treatment.

Overall, although the topic is of interest, I do not think the present manuscript provides sufficiently reliable evidence to support its central conclusions. The main problems are not merely editorial or presentational; they are methodological and affect the validity of the study’s inference. In my view, these issues are substantial enough that they cannot be resolved by routine revision alone within the current analytical framework. For these reasons, I recommend rejection.

To make this work potentially publishable in the future, the authors would need to substantially redesign the analysis, including at minimum: appropriate time-dependent or landmark modeling, adjustment for baseline hematologic values, more rigorous handling of treatment heterogeneity and supportive-care interventions, clarification of regimen-specific toxicity effects, and a more cautious interpretation of predictive performance and clinical applicability.

Reviewer #2: Please see annotated comments.

The authors should indicate in the methodology, the exact time points during after treatment, when laboratory data were collected. After how many weeks following completion of treatment were values collected ?.

The authors should provide a justification for recoding the absolute white blood cell count and not taking into consideration, the differential counts.

Reviewer #3: In this work, the authors have evaluated the utility of a novel three-lineage cytopenia score, which incorporates leukopenia, anemia, and thrombocytopenia scores as a predictor of patient prognosis. The three-lineage cytopenia score has been shown to be a strong predictor of overall survival, loco-regional recurrence-free survival, and distant metastasis-free survival, and could complement and improve the prognostic accuracy of TNM staging. This simple score is a promising tool for the accurate prediction of patient prognosis to guide personalized treatment strategies.

Overall, this retrospective study is well-designed, and rigorous statistical methods were applied to reach the main conclusions. However, the following issues should be addressed to improve the clarity of the manuscript:

1.Provide the cut-off points used for grading the single lineage cytopenias.

2.Report the percent of patients who died, had recurrence, and developed distant metastasis, along with the counts.

3.Improve the reporting of results by citing a specific figure panel containing particular data, rather than giving only general

statements.

**********

what does this mean?). If published, this will include your full peer review and any attached files.

If you choose “no”, your identity will remain anonymous but your review may still be made public.

**Do you want your identity to be public for this peer review?**  For information about this choice, including consent withdrawal, please see our Privacy Policy

Reviewer #1: No

Reviewer #2: No

Reviewer #3: **Yes:**  Geofrey Filbert Soko

**********

[NOTE: If reviewer comments were submitted as an attachment file, they will be attached to this email and accessible via the submission site. Please log into your account, locate the manuscript record, and check for the action link "View Attachments". If this link does not appear, there are no attachment files.]

To ensure your figures meet our technical requirements, please review our figure guidelines: https://journals.plos.org/plosone/s/figures

You may also use PLOS’s free figure tool, NAAS, to help you prepare publication quality figures: https://journals.plos.org/plosone/s/figures#loc-tools-for-figure-preparation.

NAAS will assess whether your figures meet our technical requirements by comparing each figure against our figure specifications.

Attachment Submitted filename: PONE-D-26-14203_reviewer (1).pdf

# Author comment: Author response to Decision Letter 1

**DOI:** 10.1371/journal.pone.0356005.r002

15 Jun 2026

Response to Reviewers

Manuscript ID: PONE-D-26-14203

Title: Prognostic Value of the Three-Lineage Cytopenia Score in Locally Advanced Nasopharyngeal Carcinoma: A Retrospective Cohort Study

Dear Editor and Reviewers,

We thank the Editor and the Reviewers for their thoughtful comments and constructive suggestions. We have carefully addressed all concerns and have revised the manuscript accordingly. All changes have been highlighted in yellow in the revised manuscript. Below we provide a point by point response to each comment.

Response to Reviewer #1

General comment: The manuscript addresses a clinically relevant question, but the core analytical framework contains major methodological problems.

Comment 1 (Time dependent bias / guarantee time bias):

The cytopenia score was derived from nadir values during treatment, while survival was measured from treatment initiation. This creates a risk of guarantee time bias. A landmark analysis or time dependent Cox model would be more appropriate.

Our response: We agree with the reviewer. To address guarantee time bias, we performed a landmark sensitivity analysis. The landmark time point was set at 7 months after treatment initiation (based on median treatment duration of approximately 4 months plus 90 days). Patients who had an event or were censored before this landmark were excluded, and survival analyses were repeated. The results of this landmark analysis are presented in Supplementary Table 5. The landmark analysis showed results consistent with the primary analysis, confirming the robustness of our findings. We have added a description of this analysis in the Methods section (highlighted in yellow) and cited the supplementary table.

Comment 2 (Residual confounding – baseline blood counts):

The score may simply reflect pre existing bone marrow reserve (baseline WBC, hemoglobin, platelets). The multivariable model did not adjust for baseline values.

Our response: We appreciate this important concern. We performed a sensitivity analysis by additionally adjusting for baseline white blood cell, hemoglobin, and platelet counts in the multivariate Cox models for all three endpoints. The results (presented in Supplementary Table 6) show that the three lineage cytopenia score remained strongly and independently associated with OS, LRFS, and DMFS (as a continuous variable). These findings indicate that the prognostic value of the score is not simply a reflection of baseline hematological status. The sensitivity analysis has been described in the Methods (highlighted in yellow) and the results are cited.

Comment 3 (Heterogeneity of treatment):

The therapeutic context is highly heterogeneous (different chemotherapy regimens, dose modifications, G CSF use, etc.), and the manuscript provides insufficient adjustment for these factors.

Our response: We acknowledge this limitation. The retrospective, single center design does not allow us to fully capture all treatment modifications and supportive care interventions. We have added a statement in the Discussion (highlighted in yellow) acknowledging this as a limitation and calling for future multicenter studies with more detailed treatment data.

Comment 4 (Overstated dose response relationship):

The data do not support a clear dose response relationship; the association is driven mainly by the score 3 subgroup. Pairwise comparisons between scores 0, 1, and 2 are often non significant.

Our response: We agree with the reviewer. We have revised the wording throughout the manuscript to avoid claiming a “clear dose response relationship” or “progressive stepwise decrease”. Instead, we now state that the association is most pronounced for patients with a score of 3 (involvement of all three lineages), and that differences between scores 0, 1, and 2 are either non significant or inconsistent across endpoints. These changes have been highlighted in yellow in the Results section (Three lineage cytopenia score and prognosis).

Comment 5 (Poor prognostic model performance and AUC <0.5 for TNM model):

The model achieved an AUC of only 0.593, and the TNM only model had an AUC below 0.5, raising concerns about model specification.

Our response: We agree that the discriminatory ability of our model is modest (AUC=0.593). We have revised the Results accordingly, stating that the model shows “modest discriminatory ability”. Regarding the TNM only model, we found that TNM staging alone performed poorly in our cohort (AUC <0.5), possibly due to sample characteristics. Therefore, we decided to remove the formal comparison with the TNM model and only present the ROC curve for our new model. The revised Figure 3B now shows only the ROC curve for the model incorporating the three lineage cytopenia score, and the corresponding text has been modified (highlighted in yellow). We also added a sentence in the Discussion acknowledging that the AUC leaves room for improvement.

Comment 6 (Unstable effect estimates, wide confidence intervals):

The hazard ratios for LRFS and DMFS in the score 3 group have extremely wide confidence intervals (e.g., 2.567–149.582).

Our response: We agree that the wide confidence intervals reflect the limited number of events, especially for LRFS (65 events). We have added a note in the Results (pairwise comparisons) and in the Discussion (limitations) to caution against overinterpretation of these estimates. These additions are highlighted in yellow.

Response to Reviewer #2

Comment 1 (Timing of laboratory data collection):

The authors should indicate the exact time points when laboratory data were collected after treatment.

Our response: We have added the following statement to the Methods section (highlighted in yellow): “Complete blood counts were measured at least weekly during radiotherapy and chemotherapy cycles. The nadir values were recorded as the lowest counts during the entire treatment period.”

Comment 2 (Use of absolute WBC count instead of differential counts):

The authors should provide a justification for using absolute white blood cell count rather than differential counts.

Our response: We have added a justification in the Methods (highlighted in yellow): “The total white blood cell count was chosen because it reflects overall leukocyte suppression, which is clinically relevant for myelosuppression grading. Differential counts were not analyzed to avoid multiple comparisons and because the three lineage cytopenia score is intended as a simple, practical tool using routine complete blood count parameters.”

Response to Reviewer #3

Comment 1 (Provide cut off points for single lineage cytopenias):

The authors should provide the cut off values used for grading leukopenia, anemia, and thrombocytopenia.

Our response: We have added the CTCAE version 5.0 cut off points in the Methods section (highlighted in yellow). These include the exact numerical thresholds for grades 1–4 for each lineage.

Comment 2 (Report percentages of patients who died, had recurrence, and developed distant metastasis):

These percentages are already present in the Results (first paragraph of Survival Outcomes).

Our response: We confirm that the percentages are already reported (e.g., “145 patients died (25.2%)”). No additional change was needed.

Comment 3 (Improve reporting of results by citing specific figure panels):

The authors should cite specific figure panels (e.g., Figure 1A–C) rather than giving only general statements.

Our response: We have revised the Results section to cite specific figure panels throughout. For example, “Figure 1A–C” instead of “Figure 1”. All changes are highlighted in yellow.

Additional Revisions (Editor’s requests)

1. Style requirements: The manuscript has been formatted according to PLOS ONE’s style templates.

2. Ethics statement: The ethics statement appears only in the Methods section (as required).

3. Data availability statement: We have updated the Data Availability Statement in the submission system as follows: “Due to ethical restrictions… data are available upon reasonable request from the corresponding author with approval from the ethics committee.” The statement is also included in the manuscript.

4. Financial disclosure role of funders: We have added the following sentence to the cover letter: “The funders had no role in study design, data collection and analysis, decision to publish, or preparation of the manuscript.”

5. Supporting Information captions: Captions for all supplementary tables have been added at the end of the manuscript.

6. Landmark and baseline adjusted sensitivity analyses: These have been added as Supplementary Tables 5 and 6, as described above.

7. Figure 3B: We have removed the TNM only ROC curve and now present only the curve for our model, with updated text and legend.

We believe that the revised manuscript has been substantially improved and addresses all concerns raised by the reviewers. We thank the Editor and Reviewers for their time and valuable input. We look forward to your favorable decision.

Sincerely,

Dong Yang, MD

Corresponding author

Attachment Submitted filename: Response to Reviewers.docx

# Peer review: Decision Letter 1

**DOI:** 10.1371/journal.pone.0356005.r003

**Authors:** Satyajeet Rath

24 Jul 2026

Dear Dr. yang,

Thank you for submitting your manuscript to PLOS ONE. After careful consideration, we feel that it has merit but does not fully meet PLOS ONE’s publication criteria as it currently stands. Therefore, we invite you to submit a revised version of the manuscript that addresses the points raised during the review process.

==============================

**ACADEMIC EDITOR:**  Minor revision to address some inconsistencies as duly highlighted by reviewers

==============================

Please submit your revised manuscript by Sep 07 2026 11:59PM. If you will need more time than this to complete your revisions, please reply to this message or contact the journal office at plosone@plos.org. When you're ready to submit your revision, log on to https://www.editorialmanager.com/pone/ and select the 'Submissions Needing Revision' folder to locate your manuscript file.

- A letter that responds to each point raised by the academic editor and reviewer(s). You should upload this letter as a separate file labeled 'Response to Reviewers'.
- A marked-up copy of your manuscript that highlights changes made to the original version. You should upload this as a separate file labeled 'Revised Manuscript with Track Changes'.
- An unmarked version of your revised paper without tracked changes. You should upload this as a separate file labeled 'Manuscript'.

If you would like to make changes to your financial disclosure, please include your updated statement in your cover letter. Guidelines for resubmitting your figure files are available below the reviewer comments at the end of this letter.

If applicable, we recommend that you deposit your laboratory protocols in protocols.io to enhance the reproducibility of your results. Protocols.io assigns your protocol its own identifier (DOI) so that it can be cited independently in the future. For instructions see: https://journals.plos.org/plosone/s/submission-guidelines#loc-laboratory-protocols. Additionally, PLOS ONE offers an option for publishing peer-reviewed Lab Protocol articles, which describe protocols hosted on protocols.io. Read more information on sharing protocols at https://plos.org/protocols?utm_medium=editorial-email&utm_source=authorletters&utm_campaign=protocols.

As the corresponding author, your ORCID iD is verified in the submission system and will appear in the published article. PLOS supports the use of ORCID, and we encourage all coauthors to register for an ORCID iD and use it as well. Please encourage your coauthors to verify their ORCID iD within the submission system before final acceptance, as unverified ORCID iDs will not appear in the published article. *Only*  the individual author can complete the verification step; PLOS staff *cannot*  verify ORCID iDs on behalf of authors.

We look forward to receiving your revised manuscript.

Kind regards,

Satyajeet Rath

Academic Editor

PLOS One

Journal Requirements:

If the reviewer comments include a recommendation to cite specific previously published works, please review and evaluate these publications to determine whether they are relevant and should be cited. There is no requirement to cite these works unless the editor has indicated otherwise.

Please review your reference list to ensure that it is complete and correct. If you have cited papers that have been retracted, please include the rationale for doing so in the manuscript text, or remove these references and replace them with relevant current references. Any changes to the reference list should be mentioned in the rebuttal letter that accompanies your revised manuscript. If you need to cite a retracted article, indicate the article’s retracted status in the References list and also include a citation and full reference for the retraction notice.

Additional Editor Comments:

Minor revision to address some inconsistencies as duly highlighted by reviewers

[Note: HTML markup is below. Please do not edit.]

Reviewers' comments:

Reviewer's Responses to Questions

**Comments to the Author**

Reviewer #1: All comments have been addressed

Reviewer #2: All comments have been addressed

Reviewer #3: All comments have been addressed

**********

2. Is the manuscript technically sound, and do the data support the conclusions?

Reviewer #1: Yes

Reviewer #2: Yes

Reviewer #3: Yes

**********

3. Has the statistical analysis been performed appropriately and rigorously?

Reviewer #1: Yes

Reviewer #2: Yes

Reviewer #3: Yes

**********

4. Have the authors made all data underlying the findings in their manuscript fully available?

The PLOS Data policy

Reviewer #1: Yes

Reviewer #2: Yes

Reviewer #3: Yes

**********

5. Is the manuscript presented in an intelligible fashion and written in standard English?

Reviewer #1: Yes

Reviewer #2: Yes

Reviewer #3: Yes

**********

Reviewer #1: Dear Authors,

Thank you for your careful revision of the manuscript. The revised version has improved substantially, and several important methodological concerns raised in the previous round have been addressed, including the potential guarantee-time bias through a landmark sensitivity analysis and residual confounding by baseline blood counts through additional adjusted analyses.

However, several issues remain and should be addressed before the manuscript can be considered suitable for publication.

1. Inconsistency regarding the ROC analysis and TNM-only model

The response letter states that the formal comparison with the TNM-only model was removed because the TNM-only model showed an AUC below 0.5. However, the Abstract and parts of the manuscript still contain statements suggesting improved discrimination versus TNM staging alone. Given the modest AUC of 0.593 and the problematic performance of the TNM-only model, claims regarding incremental predictive value should be further toned down or removed. The manuscript should consistently state that the model showed only modest discriminatory ability.

2. Overstatement of clinical utility

Although the three-lineage cytopenia score appears to be associated with survival outcomes, the discriminatory performance of the model remains modest. In addition, some estimates, particularly for score 3, have wide confidence intervals. Therefore, statements such as “strong predictive value,” “improve prognostic accuracy,” or “guide personalized treatment” should be revised to more cautious wording. The conclusions should emphasize association rather than immediate clinical implementation.

3. Reporting of sensitivity analyses

The addition of the landmark analysis and the baseline blood count-adjusted analysis is appreciated. However, these analyses should be reported more transparently. Please provide sufficient details, including the number of patients excluded before the landmark time point, the number of patients included in each analysis, the number of events, HRs, 95% CIs, and P values. These details are necessary for readers to assess the robustness of the findings.

4. Treatment heterogeneity and supportive care

The manuscript acknowledges treatment heterogeneity as a limitation, but this issue remains important because cytopenia may be influenced by chemotherapy regimen, treatment intensity, dose reduction, treatment interruption, G-CSF use, transfusion, and other supportive care. If available, these variables should be summarized and, where possible, incorporated into sensitivity or adjusted analyses. If such data are unavailable, the limitation should be described more explicitly.

5. Consistency between the response letter and revised manuscript

Please carefully check the manuscript to ensure that all revisions described in the response letter are fully reflected in the Abstract, Methods, Results, Discussion, figure legends, and supplementary material.

Overall, the manuscript has improved and may become acceptable after these minor but important revisions.

Reviewer #2: I would like to congratulate the authors on the substantial improvements made to the manuscript. The revised version addresses the major methodological and interpretative concerns raised during the initial review in a thorough and thoughtful manner.

I commend the authors for their careful and constructive responses to the reviewers' comments and wish them success with the publication of this work.

Reviewer #3: The authors should integrate the results of landmark analysis in the main results of the manuscript, rather than only citing them in the methods and discussion sections and relegating them to supplementary materials.

**********

what does this mean?). If published, this will include your full peer review and any attached files.

If you choose “no”, your identity will remain anonymous but your review may still be made public.

**Do you want your identity to be public for this peer review?**  For information about this choice, including consent withdrawal, please see our Privacy Policy

Reviewer #1: No

Reviewer #2: No

Reviewer #3: No

**********

[NOTE: If reviewer comments were submitted as an attachment file, they will be attached to this email and accessible via the submission site. Please log into your account, locate the manuscript record, and check for the action link "View Attachments". If this link does not appear, there are no attachment files.]

To ensure your figures meet our technical requirements, please review our figure guidelines: https://journals.plos.org/plosone/s/figures

You may also use PLOS’s free figure tool, NAAS, to help you prepare publication quality figures: https://journals.plos.org/plosone/s/figures#loc-tools-for-figure-preparation.

NAAS will assess whether your figures meet our technical requirements by comparing each figure against our figure specifications.

# Author comment: Author response to Decision Letter 2

**DOI:** 10.1371/journal.pone.0356005.r004

25 Jul 2026

Response to Reviewers

Manuscript ID: PONE-D-26-14203R1

Title: Prognostic Value of the Three-Lineage Cytopenia Score in Locally Advanced Nasopharyngeal Carcinoma: A Retrospective Cohort Study

Dear Editor and Reviewers,

We sincerely thank the Academic Editor and the Reviewers for their thoughtful and constructive comments on our revised manuscript. We are grateful for the positive feedback and the recognition that our previous revisions have substantially improved the manuscript. We have now carefully addressed all remaining concerns raised in this round of review. All changes have been highlighted in yellow in the revised manuscript. Below we provide a point-by-point response to each comment.

Response to Reviewer #1

General comment: The authors have addressed the major methodological concerns, but several issues remain.

Comment 1 (Inconsistency regarding ROC analysis and TNM-only model):

The response letter states that the formal comparison with the TNM-only model was removed because the TNM-only model showed an AUC below 0.5. However, the Abstract and parts of the manuscript still contain statements suggesting improved discrimination versus TNM staging alone. Given the modest AUC of 0.593 and the problematic performance of the TNM-only model, claims regarding incremental predictive value should be further toned down or removed.

Our response: We thank the reviewer for this careful observation. We have revised the Abstract to remove any suggestion of comparison with the TNM-only model. The Abstract now states that the model yielded an AUC of 0.593 (95% CI: 0.533–0.652, P=0.001), indicating modest discriminatory ability, without claiming superiority over TNM staging. Similarly, in the Results section (3.4.1), we have modified the concluding sentence to state that the score “may provide additional prognostic information,” while acknowledging that the modest AUC indicates further refinement is needed. In the Discussion, we have changed “significantly improved” to “showed modest improvement.” All changes are highlighted in yellow.

Comment 2 (Overstatement of clinical utility):

Although the three-lineage cytopenia score appears to be associated with survival outcomes, the discriminatory performance remains modest, and some estimates have wide confidence intervals. Statements such as “strong predictive value,” “improve prognostic accuracy,” or “guide personalized treatment” should be revised to more cautious wording.

Our response: We agree with the reviewer. We have revised the Abstract, Discussion, and Conclusion throughout to adopt more cautious wording. Specifically:

• In the Abstract, “strong predictive value” has been replaced with “promising predictive value,” and “improve prognostic accuracy and guide personalized treatment” has been changed to “serve as a useful adjunct to traditional staging for risk stratification.”

• In the Discussion (clinical implications paragraph), we have removed “effective” and changed “should consider” to “may consider,” adding that “prospective validation is needed before clinical implementation.”

• In the Conclusion, “strong predictive value” has been changed to “promising predictive value,” “enhances prognostic accuracy” to “may provide additional prognostic information,” and “valuable tool for personalized treatment” to “potential tool for risk stratification.”

All changes are highlighted in yellow.

Comment 3 (Reporting of sensitivity analyses):

The addition of the landmark analysis and baseline blood count-adjusted analysis is appreciated. However, these analyses should be reported more transparently, including the number of patients excluded, the number of patients included, events, HRs, 95% CIs, and P values.

Our response: We have added a new paragraph in the Results section (Survival Outcomes) reporting the landmark analysis results in detail. Specifically, we now state that 36 patients (6.3%) were excluded from the OS analysis, leaving 540 patients with 82 events, and that the three-lineage cytopenia score (as a continuous variable) remained significantly associated with OS (HR=2.182 per 1 point increase; 95% CI: 1.741–2.734; P<0.001), with consistent results for LRFS and DMFS. Full details are also provided in Supplementary Table 5. This change is highlighted in yellow.

Comment 4 (Treatment heterogeneity and supportive care):

Treatment heterogeneity remains an important issue because cytopenia may be influenced by chemotherapy regimen, treatment intensity, dose reduction, G CSF use, transfusion, and other supportive care. These variables should be summarized or, if unavailable, the limitation should be described more explicitly.

Our response: We acknowledge that detailed information on G CSF use, transfusion support, and chemotherapy dose modifications was not uniformly available in this retrospective dataset. We have added a clear statement to this effect in the Limitations paragraph of the Discussion (highlighted in yellow). We also note that these unmeasured factors may have influenced the observed associations and emphasize the need for future prospective studies with comprehensive treatment and supportive care documentation.

Comment 5 (Consistency between the response letter and revised manuscript):

Please carefully check the manuscript to ensure that all revisions described in the response letter are fully reflected in the Abstract, Methods, Results, Discussion, figure legends, and supplementary material.

Our response: We have carefully reviewed the entire manuscript and confirmed that all revisions described in our previous response letter have been incorporated. We have also verified that the Abstract, Methods, Results, Discussion, figure legends, and supplementary materials are internally consistent and accurately reflect the changes made.

Response to Reviewer #2

General comment: The manuscript has been substantially improved.

Our response: We sincerely thank the reviewer for the positive feedback and for acknowledging the improvements made to the manuscript. We are grateful for the reviewer’s time and thoughtful evaluation.

Response to Reviewer #3

General comment: The authors should integrate the results of landmark analysis in the main results of the manuscript, rather than only citing them in the methods and discussion sections and relegating them to supplementary materials.

Our response: We agree with the reviewer. We have now integrated the landmark analysis results into the main Results section. A new paragraph has been added in the Survival Outcomes section (highlighted in yellow), which reports the key findings of the landmark sensitivity analysis, including the number of patients excluded, the number of patients included and events, and the HRs with 95% CIs and P values for all three endpoints. The detailed results remain available in Supplementary Table 5 for readers who wish to examine the complete data.

Additional Revisions

In addition to the reviewer-specific changes, we have made the following adjustments:

• In the Discussion (first paragraph, point 3), we changed “significantly improved” to “showed modest improvement” to better reflect the modest AUC.

• In the Results section (3.4.1), we revised the concluding sentence to state that the score “may provide additional prognostic information” while acknowledging the modest AUC indicates further refinement is needed.

• The Abstract has been revised to remove any comparison with the TNM-only model and to adopt more cautious wording regarding predictive value and clinical utility.

We believe that the revised manuscript has been further strengthened and now addresses all remaining concerns. We are grateful for the reviewers’ time, expertise, and constructive suggestions, which have significantly improved our work. We look forward to your favorable decision.

Sincerely,

Dong Yang, MD

Corresponding author

Attachment Submitted filename: Response_to_Reviewers_auresp_2.docx

# Editor report: Decision Letter 2

**DOI:** 10.1371/journal.pone.0356005.r005

**Authors:** Satyajeet Rath

28 Jul 2026

Prognostic Value of the Three-Lineage Cytopenia Score in Locally Advanced Nasopharyngeal Carcinoma: A Retrospective Cohort Study

PONE-D-26-14203R2

Dear Dr. yang,

We’re pleased to inform you that your manuscript has been judged scientifically suitable for publication and will be formally accepted for publication once it meets all outstanding technical requirements.

Within one week, you’ll receive an e-mail detailing the required amendments. When these have been addressed, you’ll receive a formal acceptance letter and your manuscript will be scheduled for publication.

An invoice will be generated when your article is formally accepted. Please note, if your institution has a publishing partnership with PLOS and your article meets the relevant criteria, all or part of your publication costs will be covered. Please make sure your user information is up-to-date by logging into Editorial Manager at Editorial Manager® and clicking the ‘Update My Information' link at the top of the page. For questions related to billing, please contact billing support.

If your institution or institutions have a press office, please notify them about your upcoming paper to help maximize its impact. If they’ll be preparing press materials, please inform our press team as soon as possible -- no later than 48 hours after receiving the formal acceptance. Your manuscript will remain under strict press embargo until 2 pm Eastern Time on the date of publication. For more information, please contact onepress@plos.org.

Kind regards,

Satyajeet Rath

Academic Editor

PLOS One

Additional Editor Comments (optional):

All the queries have been duly addressed.

Reviewers' comments:

# Editor report: Acceptance letter

**DOI:** 10.1371/journal.pone.0356005.r006

**Authors:** Satyajeet Rath

PONE-D-26-14203R2

PLOS One

Dear Dr. yang,

I'm pleased to inform you that your manuscript has been deemed suitable for publication in PLOS One. Congratulations! Your manuscript is now being handed over to our production team.

At this stage, our production department will prepare your paper for publication. This includes ensuring the following:

* All references, tables, and figures are properly cited

* All relevant supporting information is included in the manuscript submission,

* There are no issues that prevent the paper from being properly typeset

You will receive further instructions from the production team, including instructions on how to review your proof when it is ready. Please keep in mind that we are working through a large volume of accepted articles, so please give us a few days to review your paper and let you know the next and final steps.

Lastly, if your institution or institutions have a press office, please let them know about your upcoming paper now to help maximize its impact. If they'll be preparing press materials, please inform our press team within the next 48 hours. Your manuscript will remain under strict press embargo until 2 pm Eastern Time on the date of publication. For more information, please contact onepress@plos.org.

You will receive an invoice from PLOS for your publication fee after your manuscript has reached the completed accept phase. If you receive an email requesting payment before acceptance or for any other service, this may be a phishing scheme. Learn how to identify phishing emails and protect your accounts at https://explore.plos.org/phishing.

If we can help with anything else, please email us at customercare@plos.org.

Thank you for submitting your work to PLOS ONE and supporting open access.

Kind regards,

PLOS ONE Editorial Office Staff

on behalf of

Dr. Satyajeet Rath

Academic Editor

PLOS One
